# A comparative assessment of cluster-based regionalization approaches using conceptual rainfall–runoff models

**DOI:** 10.1038/s41598-026-49424-z

**Published:** 2026-04-27

**Authors:** Jamal Hassan Ougahi, John S. Rowan

**Affiliations:** 1 UNESCO Centre of Water Law, Policy & Science, University of Dundee, Dundee, UK; 2 Higher Education Department, Government of the Punjab, Lahore, Pakistan

**Keywords:** Hydrological models, Clustering, Regionalization methods, Support vector regression, Ungauged catchments, Climate sciences, Environmental sciences, Hydrology

## Abstract

Accurate streamflow prediction in ungauged catchments remains a central challenge in hydrology. We investigated the utility of regionalization methods to explore how hydrologically informed clustering influences parameter transferability and performance of different runoff models applied over a national scale. This study evaluates the performance of conceptual hydrological models (GR4J, GR6J, HBV) using support vector regression (SVR), *k*-nearest neighbours and weighted average parameter (WAP) regionalization approaches across catchments in the CAMELS-GB dataset. The HBV model performed best in montane northern Britain where snow melt contributes to seasonal flows but otherwise struggled to capture the complexity of urbanised and agriculturally modified lowlands in southern and eastern Britain where more process-explicit and parameterised GR4J and GR6J models excelled in performance. Our results demonstrate that SVR provides a robust and flexible regionalization framework by effectively capturing nonlinear catchment–response relationships to outperform traditional similarity-based approaches across most hydrological regimes. Clustering also enhanced WAP regionalization for GR4J and GR6J, whereas HBV remained largely unaffected due to its higher parameter dimensionality and internal compensation. Our findings reveal that parameter transferability is strongly cluster-dependent and regionalization strategies must explicitly account for interactions between model structure, regionalization method, and dominant regional hydrological characteristics. The significance of the contribution lies in the generalizability of the approach to ungauged basins where robust flow predictions are required for water resource management, risk assessment and climate change adaptation studies worldwide.

**Supplementary Information:**

The online version contains supplementary material available at 10.1038/s41598-026-49424-z.

## Introduction

Rainfall–runoff models play a vital role in supporting decision-making processes including water resource management, flood prediction and drought warnings^1,2^. Over the past decades, conceptual hydrological models have become essential tools for streamflow prediction and water resource management (Boughton and Droop^3^) including HBV^4^, Sacramento (Burnash et al.^5^), GR4J^6^, GR6J^7^, PRMS (Leavesley et al.^8^), and TOPMODEL^9^. Accurate process representation in such models relies on robust model calibration^10,11^ or regionalization^12^, high quality input data^13^ and improved model structures^14^. These advances have produced a wide range of models and configurations, but no single approach demonstrates consistent superiority^15^. Parameter regionalization is challenging in complex models with many interdependent parameters that weaken parameter–catchment relationships and require large datasets and substantial effort (Guo et al., 202). Therefore, most studies rely on simpler conceptual models with fewer, more independent parameters to reduce these issues and improve regionalization performance typically exploiting aspects of basin similarity^16–19^.

Parameter regionalization is widely used approach for transferring calibrated model parameters from gauged to ungauged catchments at the basin scale^20,21^. However, robust predictions based on parameter regionalization have long-suffered from the limitations imposed by sparse and short duration flow records^22–24^. Recently, the availability of large-sample hydrological data from many regions, such as the Catchment Attributes and Meteorology for Large-sample Studies (CAMELS) for United States, CAMELS-GB for the Great Britain (Coxon et al.^25^), CAMELS-AUS for Australia and CAMELS-CL for Chile have provided increased interest in transferring data-driven hydrological insights across catchments and regions^26^. New levels of data availability have been accompanied by the emergence of machine learning (ML) approaches which offer the prospect of state-of-the-art modelling performance^27^. Catchment attributes such as ecoregion setting, topography, regional climate land use/land cover influence hydrological behaviour (e.g., seasonality of precipitation, flashiness and nature of baseflow characteristics) presenting each catchment as a unique prediction challenge in relation to hydrological model performance^28^. Using the explanatory potential of a regional pool of ‘donor catchments’ mitigates reliance on weaker analogues and enhances the likelihood of isolating the most hydrologically similar catchments and most robust flow predictions^29^.

Hydrological models are fundamental for accurately estimating catchment runoff and play a vital role in managing water resources and issuing flood and drought warnings^17^. Hydrological models coupled with regionalization methods provide a reliable approach for predicting streamflow in ungauged watersheds, especially in data-scarce regions^30^. The GR4J model has been widely used in hydroclimate change research worldwide and has shown strong performance across a variety of catchments in the UK^31,32^, (Smith et al.^33^). The simplicity and robust performance of GR4J model for predicting streamflow is model widely used for parameter transfer in hydrological regionalization^30,34^, (Ajmal et al.^35^). The effectiveness of regionalization methods varies with hydrological model complexity (GR4J, WASMOD, HBV, and XAJ), climatic conditions, and regional characteristics, with more complex models (e.g., XAJ) performing better under stationary conditions but showing larger declines under nonstationary climates^18,19^. Many studies suggest that the effectiveness of regionalization methods depends both on the choice of model and the characteristics of the study area (e.g^12,36,37^.,). In contrast, some studies have reported that differences in hydrological model had little impact on runoff prediction in ungauged basins^38,39^. The effectiveness of a regionalized hydrological model depends on both the flow regime and the model structure, with GR4J better capturing high-flow dynamics and GR6J better representing low-flow conditions^40^. In a recent study of 200 catchments across the UK, GR4J demonstrated good performance across most catchments for general evaluation metrics, whereas GR6J showed slightly better performance in simulating low-flow conditions^31^. In Ireland, regionalization of hydrological models showed that GR4J coupled with Random Forests performs best for high flows, while GR6J performs best for low flows, highlighting the importance of model choice, objective function, and parameter-linking method for successful prediction in ungauged catchments^40^. GR4J demonstrated the most robust performance for streamflow prediction in semi-arid and humid catchments, outperforming more complex models due to its simpler structure^41^. Ensemble streamflow predictions were skilful across most UK catchments using the GR4J model with forecast skill highest at short lead times and gradually declining with increasing lead time^32^.

Over the past two decades, numerous studies have explored and compared different regionalization approaches (e.g^30^.). These approaches are typically classified into two main categories such as regression-based He et al.^42^^43^, and similarity-based methods (Oudin et al.^44^). Each regionalization method exhibits unique strengths and performance characteristics, making it difficult to identify universally optimal regionalization strategy. The regression-based method examines complex non-linear relationships between basin attributes and calibrated parameters to estimate parameters for ungauged basins^45,46^. This approach often faces limitations due to the loss of parameter interactions and equifinality^40,47^, (Oudin et al.^44^). One of the widely used regression-based methods is multivariate linear regression (MLR) wherein a linear regression model is assumed to sufficiently describe the relationship between model parameters and watershed properties^48,49^. Young^50^ reported that regression-based regionalization outperformed the spatial proximity method in the UK. In the UK, regression-based regionalization of HBV model parameters did not outperform default values, indicating limitations of simple parameter-transfer approaches in ungauged catchments^51^. Recently, most of studies have implemented non-linear and ML based algorithms such as artificial neural networks (ANN), random forests (RF), support vector machines (SVM) etc. to infer hydrological relationships^52,53^, (Ougahi and Rowan^54^). However, no single regionalization approach consistently outperforms others^55^.

Cluster-based regionalization methods have received increasing attention to account for hydrological similarity while helping to address the challenges of classifying catchments without extensive prior analysis^17,56^. Hu et al.^57^ reported that the simulation performance of linear regression‐based method is largely improved by donor catchment clustering. They grouped (or *clustered*) the donor catchments based on their similar characteristics before applying the regression-based regionalization which improved the performance of a model significantly. Parameter transfer between dissimilar catchments can introduce significant errors which can be mitigated by grouping catchments with similar hydrological characteristics^57^. Therefore, clustering catchments based on expected hydrological similarity and transferring model parameter sets within each cluster is considered more practical^55^.

The number of regionalization methods and models used in prior studies remains too limited to draw general conclusions. Few studies have evaluated regionalization methods using multiple hydrological models, but results indicate consistent performance across models of different complexity, for example, SIMHYD and XAJ in Australia (Li and Zhang^58^) and GR4J and SIMHYD in the southeast Tibetan Plateau (Li et al.^59^). The model complexity and the number of parameters alone do not reliably predict its performance^41^. A more comprehensive study is needed to assess how regionalization performance varies across multiple hydrological models of different complexity based on clustering approach particularly under temperate and year-round precipitation conditions. The performance of regionalization method depends on both the study area and the choice of hydrological model^12^. Most of these investigations are limited to a single model applied in a specific region, with only a few assessing regionalization across multiple models^18,19,28,60^. In well-characterized yet hydrologically diverse catchments, it is essential to conduct a detailed assessment of hydrological model performance and regionalization methods to investigate the influence of catchment clustering and model structure on model prediction skill.

This study presents a large-sample evaluation of regionalization strategies across hydrologically diverse UK catchments using the CAMELS-GB dataset. It further explores and extends regionalization approaches including non-linear regression using Support Vector Regression (SVR), as well as methods based on spatial proximity and physiographic climatic similarity. We systematically compared multiple hydrological model structures alongside alternative regionalization approaches to understand whether a specific regionalization method is robust across model types. Specifically, we evaluated how catchment regimes or clusters, defined by their similarities, enable effective model generalization and reliable parameter transfer. By examining performance variability across diverse catchment regimes or clusters, this research provides mechanistic insight into why and when regionalization succeeds or fails. The large-sample design ensures statistical generalisability, enabling the development of practical guidance applicable to both data-rich and data-scarce catchments. In doing so, the study advances regionalization theory while delivering operationally relevant recommendations for improving prediction in ungauged basins under diverse hydroclimatic conditions. These insights can inform hydrological modelling strategies in data-limited or ungauged regions worldwide by using robust regionalization methods for improving streamflow prediction to support risk-informed water resource management (cf^61,62^..

## Materials and methods

**Fig. 1:**
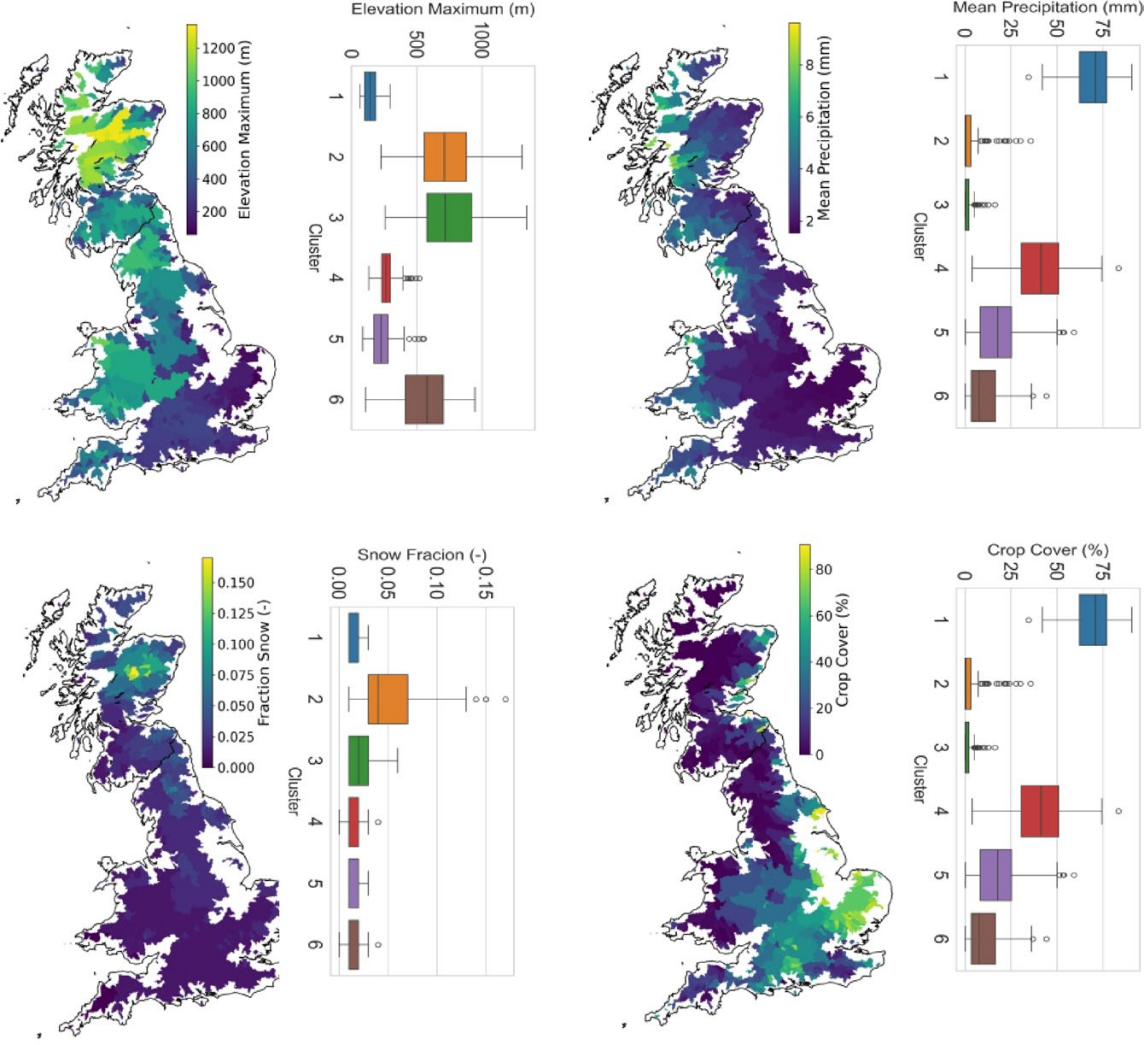
Topography, land cover and climate characteristics of Great Britain (GB) in the studied catchments and hydrologically similar group of catchments represented by six clusters.

Figure 1 illustrates the distribution of 664 study catchments, topography, land cover and climate characteristics of Great Britain (GB). Large-sample machine-learning (ML) models can learn generalized relationships between catchment characteristics and hydrological model parameters by training on data from hundreds of gauges simultaneously^2,63^. This generally enables more reliable parameter predictions for ungauged catchments compared to single-site approaches. Here, we used CAMELS-GB (Catchment Attributes and MEteorology for Large-sample Studies for GB) data comprising hydrometeorological time series and landscape attributes for a large-sample of catchments publicly available^64^. Here we included 15 static catchment descriptors from the CAMELS-GB dataset (Table S2). Catchment descriptors were selected based on their established importance in runoff generation and regionalization studies, including topographic (e.g., elevation, slope), land cover, and long-term climatic indices. To ensure statistical robustness, we conducted correlation analysis to identify highly correlated variables and retained one representative descriptor from each correlated group, thereby reducing redundancy and mitigating multicollinearity in subsequent clustering and regionalization analyses. This dataset provides attributes for 664 catchments across GB from the UK National River Flow Archive together with a suite of meteorological timeseries (https://www.earth-system-science-data.net/). The catchment descriptors were used to group catchments into clusters.

**Fig. 2:**
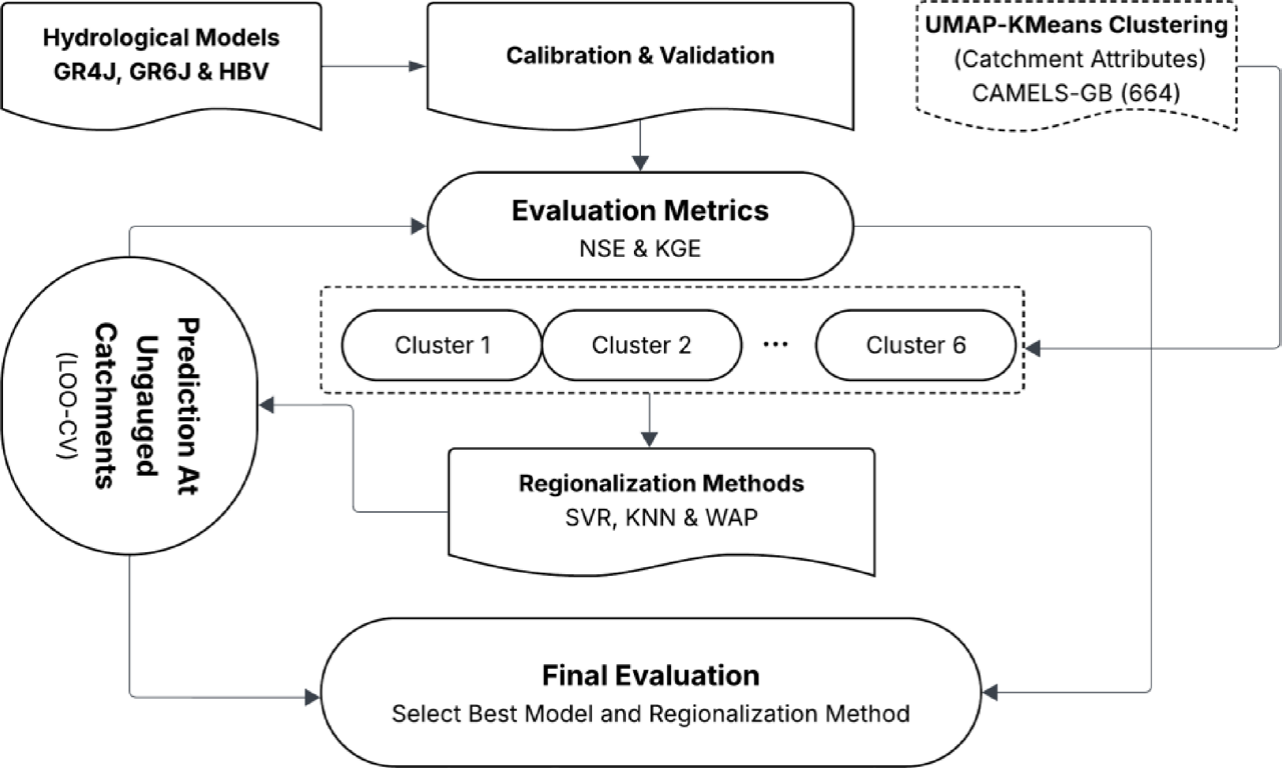
Methodological framework and workflow for model calibration and comparison, catchment clustering and regionalization approaches to parameter transfer to ungauged flow predictions.

Figure 2 presents the workflow of our novel ML enabled regionalised streamflow forecasting approach. The process begins with calibrating three hydrological models across 664 gauged catchments. Then catchment attributes are used to classify these basins into six hydrologically similar groups (Clusters). Three regionalization methods were applied within each cluster using leave-one-out cross-validation (LOOCV) to evaluate performance of hydrological models at ungauged basins.

### Clustering

**Fig. 3:**
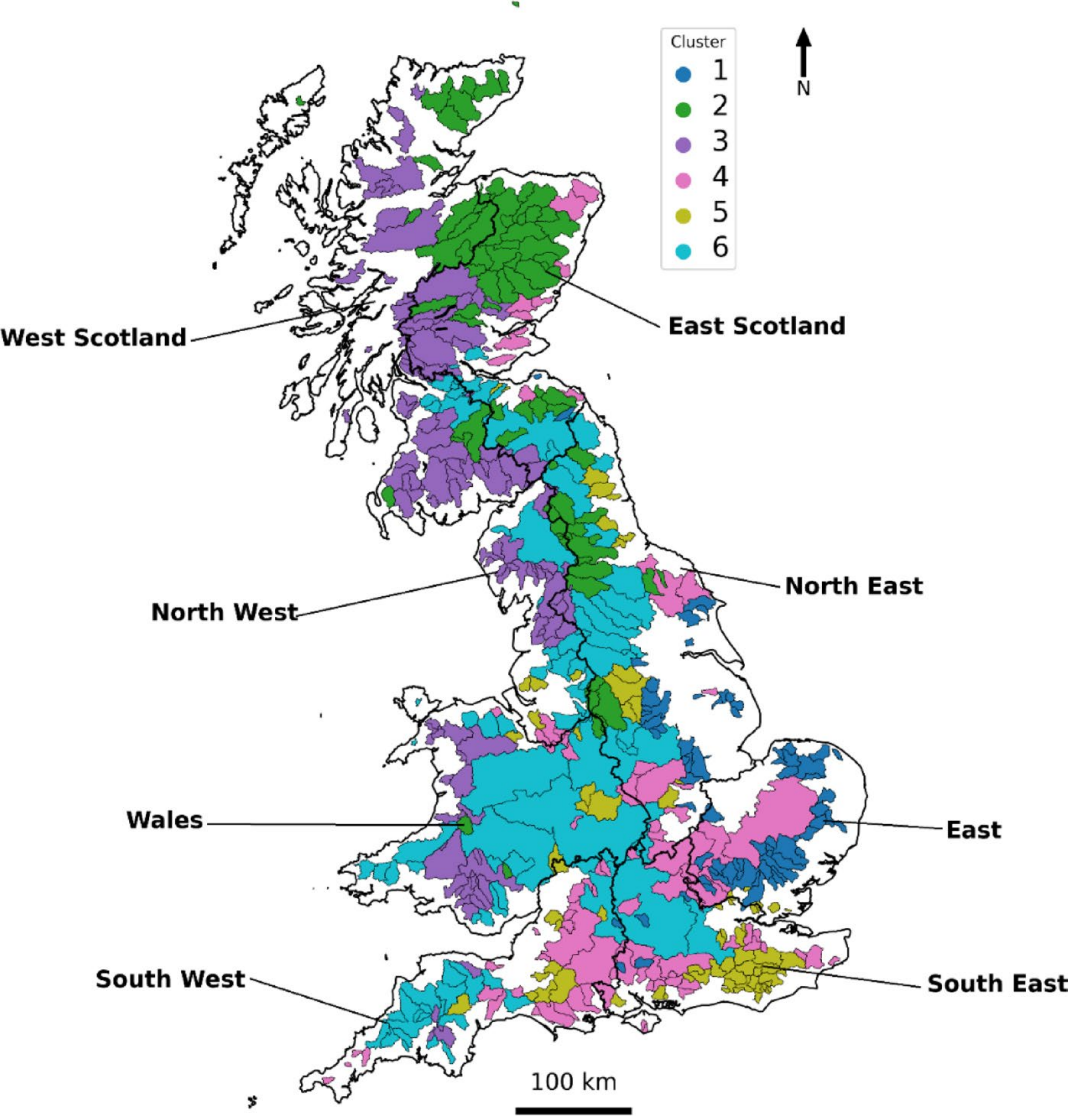
Distribution and extent of six ‘hydrologically-similar’ clusters derived from the CAMELS-GB inventory of catchments (n = 664) with the spatial pattern largely reflecting regional climate, physiography, continentality and land use & land cover [LULC] differences. Note Great Britain is the UK’s largest contiguous land mass (omitting Northern Ireland and most offshore islands).

Clustering is an unsupervised learning technique which groups unlabelled data into clusters with high intra-cluster similarity and low inter-cluster similarity (Jain et al.^65^). UMAP (Uniform Manifold Approximation and Projection) is a dimensionality reduction technique that can be used to project high-dimensional data into a lower-dimensional space (usually 2D or 3D) while preserving the local and global structure of the data^66^. Catchment attributes were extracted before being standardized and reduced to two dimensions (see Table S2). Clustering in the reduced space was then performed using k-means (with (k = 6) to group hydrologically similar catchments. From the 664 catchments, six clusters were identified across GB (Fig. 3).

The methodology for determining the optimal number of clusters involves cluster validity indices (CVIs) to evaluate clustering quality^67^. Dunn’s Index is an example of an internal CVI which is calculated as the ratio of minimum inter-cluster distance to maximum intra-cluster distance with higher values indicating better clustering quality^68^. It measures how well-separated and compact clusters are by comparing the smallest distance between clusters (inter-cluster distance) to the largest distance within any cluster (intra-cluster distance).

1 $$D = min_{i} \left( {min_{j} \left( {\frac{{min_{{x \in { complement }i,y \in { complement }j}} d\left( {x,y} \right)}}{{max_{k} \left( {max_{k} \left( {max_{x,y \in ck} d\left( {x,y} \right)} \right)} \right)}}} \right)} \right)$$

where, ${c}_{i,}{c}_{j and}{c}_{k}$ are different clusters in the dataset, $d\left(x,y\right)$ distance (e.g. Euclidean) between two points x and y, ${min}_{x\in \complement i,y\in \complement j}d\left(x,y\right)$ the smallest distance between any two points with the same cluster $ck$, this represents inter cluster compactness.

This approach ensures that the number of clusters represent the optimal clustering solution. Six clusters were selected as the optimal compromise between cluster distinctiveness and robust cluster sizes (Table S4).

### Model description

We employed two parsimonious hydrological models, specifically GR4J and GR6J and a relatively complex model such as HBV. These models use spatially averaged catchment daily air temperature, precipitation and potential evapotranspiration as inputs. These models were assessed against each other to simulate daily catchment water balance processes. These are briefly described.

#### GR4J

Is a four-parameter lumped rainfall–runoff model particularly effective for low-flow estimation^6^. It has shown strong performance across a variety of catchments in the UK and worldwide^31,69^. In this model, effective precipitation is obtained by reducing total precipitation to account for vegetation interception and evapotranspiration losses from a soil-moisture accounting storage. The four free parameters (e.g., maximum capacity of the production store (X1), Groundwater potential exchange (X2), Routing store capacity (X3) and the time characteristics of the unit hydrographs (X4)) are used to calibrate the model against streamflow observations (Table 1).

**Table 1:** Hydrological model parameters for the GR4J, GR6J, and HBV models, including description, range and units used in the study.

Parameters	Model	Description	Range	Units
X1	GR4J/GR6J	Production store capacity	100–1400	mm
X2	GR4J/GR6J	Inter-catchment exchange coefficient	− 4–4	mm/d
X3	GR4J/GR6J	Routing store capacity	0–500	mm
X4	GR4J/GR6J	Unit hydrograph time constant	0–10	d
X5	GR6J	Inter-catchment exchange threshold	− 4–4	–
X6	GR6J	Coefficient for emptying exponential store	0–20	mm
CFMAX	HBV	Melting factor	1–20	mm/°C/d
TT	HBV	Threshold temperature for snowfall	− 4–4	°C
CFR	HBV	Refreezing coefficient	0–0.1	–
LP	HBV	Critical soil moisture	0.3–1	–
FC	HBV	Maximum water storage capacity	50–700	m
BETA	HBV	Shape coefficient of recharge function	1–6	–
SUMAX	HBV	Upper reservoir water level threshold	0–0.1	m
PERC	HBV	Maximum percolation	0–0.8	1/d
K0	HBV	Additional recession coefficient of upper groundwater store	0.05–0.99	1/d
K1	HBV	Recession coefficient of upper groundwater store	0.01–0.8	1/d
K2	HBV	Recession coefficient of lower groundwater store	0.001–0.15	1/d
TTSM	HBV	Threshold temperature for snowmelt	0	°C

#### GR6J

Is a daily, lumped, continuous rainfall–runoff model based on simple concepts, including the use of reservoirs and a unit hydrograph, making it comparable to classical models like HBV (Bergström^70^) and VIC (Liang et al.^71^). GR6J is a six-parameter version of the GR model suite designed to enhance the simulation of low flows and groundwater exchanges. In recent years, it has seen growing use in UK and around the world for water resource applications (e.g., Anglian Water 2021)^28^. Pushpalatha et al.^7^ evaluated GR6J against five other hydrological models across 1000 French catchments and found its performance to be competitive.

GR6J is enhanced version of GR4J model with improvements in X5 parameter that represents the time to peak (in days) of the first unit hydrograph (UH1) to reflect the delay in flow response. X6 parameter represents the threshold for groundwater exchange (dimensionless) and also acts as the exponential storage control parameter (mm), governing the rate at which water is released from storage. These advancements have enhanced the accuracy of model under low-moisture conditions^7^. The effectiveness of these improvements has been demonstrated in recent applications^72,73^. All six parameter ranges are maintained at their default lower and upper bounds, with parameters normalized during the calibration process.

#### HBV

Hydrologiska Byråns Vattenbalansavdelning (HBV) is a conceptual hydrological model which simulate daily catchment water balance processes, including snow accumulation and melt, soil moisture changes, and surface runoff^4^. It requires daily precipitation, temperature, and potential evapotranspiration as inputs. Model parameters and calibration ranges were adopted from previous studies^22^. The HBV model was selected due to its flexibility, computational efficiency, and demonstrated effectiveness across a wide range of climatic and physiographic conditions as well as its successful use in numerous regionalization studies Becket al.^74^^75^,. The model was calibrated for each catchment over the period with simultaneous observed streamflow and input data, using a lumped approach to minimize computational time. Further implementation details and comparisons with other HBV-type models can be found in Astagneau et al.^76^ and Jansen et al.^77^.

### Model calibration

Hydrological model parameters were obtained through calibration for each catchment prior to regionalization. The simulation period was divided into three parts such as warm-up, calibration, and validation. The first year (1999) was designated as the warm-up period to minimize uncertainties associated with initial conditions. The remaining time series was then split into calibration (2000–2008) and validation periods (2009–2014). Model calibration was performed using the SCE-UA automatic optimization algorithm^78^, implemented via the *sceua* function in the *SPOTPY* Python package^79^, for lumped Python implementations of the GR4J, GR6J, and HBV hydrological models. For each catchment, ten calibrated parameter sets were generated to select the best parameter set yielding the highest NSE.

The calibration process was performed using optimization based on objective function such as the Nash–Sutcliffe Efficiency (NSE). The NSE assesses overall model performance across the entire hydrograph and can be calculated as follows:

2 $$NSE = 1 - \frac{{\sum (Q_{obs} - Q_{sim} )^{2} }}{{\sum (Q_{obs} - \overline{Q}_{ibs} )^{2} }}$$

where ${Q}_{obs}$, ${Q}_{sim}$ and ${\overline{Q} }_{obs}$ are the observed, simulated and mean of observed runoff respectively.

Following calibration, model performance was evaluated using NSE and KGE, providing a comprehensive evaluation of the ability of model to reproduce the magnitude, timing, and variability of observed flows. Kling–Gupta Efficiency (KGE) was included in the evaluation to capture not only overall fit but also the correlation, bias, and variability of the simulated flows, offering a comprehensive assessment of model skill^80^.

### Regionalization methods

In hydrology, spatial proximity, physical similarity and regression are commonly used approaches for regionalization^18–20,60^. After calibration, parameters of all three hydrological models were transferred to target catchments. The model was executed using daily precipitation, potential evapotranspiration, and temperature as inputs to simulate streamflow. In regionalization methods, all catchments are used as donor catchments regardless of their performance at the calibration stage.

Physical similarity assumes that neighbouring basins have similarities in climate, soil type, land use and cover, slope, altitude, and other characteristics which lead to similar hydrological responses^41^. In the distance-based regionalization framework, two methods were implemented to transfer parameters from gauged to ungauged catchments such as *k*-nearest neighbours (*k*NN) and weighted average parameter (WAP). *k*NN is a hydrological regionalization method which estimates model parameters at an ungauged catchment by identifying the parameters from *k* most similar catchments without averaging according to a defined distance metric (e.g., Euclidean distance, Mahalanobis distance). In WAP regionalization approach, multiple donor catchments were used by assigning a weight to each catchment based on similarity to the target catchment. Both methods preserve the internal correlation structure of the parameter set which is a key factor in their superior performance^30,60^.

To identify most suitable donor, the Euclidean distance is calculated between the target and all gauged catchments using selected catchment attributes. The Euclidean distance (dist) is a metric that can express similarities (small distances) or differences (large distances) between n-attributes of two catchments (a and b) in an n-dimensional space of attributes^81^.

This Euclidean distance is calculated using Eq. (3) below.

3 $$dist\left( {a,b} \right) = \sqrt {\mathop \sum \limits_{k = 1}^{n} [atrib_{k} \left( a \right) - atrib_{k} (b)]^{2} }$$

The average parameter set transfer from multiple donors has the potential to improve results significantly over a single donor^36,82^. In WAP regionalization, the influence of donor catchments decreases smoothly as their distance or dissimilarity from the target catchment increases. This approach provides more stable predictions and reduces sensitivity to the specific choice of *k* compared to standard kNN method. Following the framework of previous studies^18,19,83^, (Oudin et al.^44^) the averaging is performed using the IDW scheme, in which the distance is either the Euclidian distance for spatial proximity or the physical similarity^36,82^. It has been shown that that IDW slightly outperforms the arithmetic mean, although the differences might not be statistically significant^84^. The closer donor basins (smaller distances) receive higher weights, and farther basins get lower weights.

The relationship between model parameters and catchment characteristics are often complex and highly non-linear^17^. To capture these complexities, we employed the Support Vector Regression (SVR) with a radial basis function (RBF) kernel to estimate hydrological model parameters for ungauged basins as a function of catchment descriptors. SVR was trained using donor catchment features as predictors and calibrated model parameters as targets.

4 $$Maximize \left( {\left[ {\frac{1}{m}\mathop \sum \limits_{i = 1}^{m} R_{i}^{2} } \right] + \left[ {\frac{{R_{a}^{2} + R_{b}^{2} + R_{c}^{2} + R_{d}^{2} }}{4}} \right]} \right)$$

where m is the number of catchment sites, ${R}_{i}^{2}$ is the simulation effect of the $i$ th site observations and where the model simulation values, ${R}_{a}^{2}+{R}_{b}^{2}+{R}_{c}^{2}+{R}_{d}^{2}$ represent the regression of model parameters and catchment attributes equation fitting effect (here, the “abcd” model has four parameters a, b, c, and d, so there are four effective regression parameters). This method captures potentially non-linear relationships between catchment descriptors and hydrological parameters, enabling more flexible parameter estimation than distance-based methods.

In this study, we used default settings (C = 1.0, ε = 0.1, and γ = ‘scale’) for training one SVR per model parameter on donor catchments within each cluster (Table S5). All input attributes were standardized prior to modelling. The target catchment was excluded to ensure strict leave-one-out cross-validation. kNN regionalization selected the nearest donor (k = 1) based on Euclidean distance in UMAP space, while WAP regionalization averaged the top three nearest donors (k = 3) using inverse-distance weighting, normalized to sum one. This framework ensures reproducible comparisons and allows assessment of which clusters or catchment regimes consistently enable reliable parameter transfer, rather than introducing new regionalization algorithms.

### The LOOCV method

A leave-one-out cross-validation (LOO-CV) procedure is commonly used to assess how well models predict streamflow under ungauged conditions for each catchment in the dataset^16,56^. In LOO-CV technique, one catchment is left out of the training set and treated as an ungauged catchment (as if no streamflow data were available for it). The model trained on the remaining catchments is then used to predict streamflow for the left-out basin. For each target catchment, hydrological model parameters were estimated using information from other catchments within the same cluster. To obtain the regionalization performance in all catchments, this process was repeated by assuming each catchment to be pseudo-ungauged in turn.

It is also worth mentioning that the performance of regionalization methods during calibration and validation periods doesn’t necessary imply the same as for the gauged catchments. In the gauged case, validation period is used to test the temporal transferability of model parameters while, the calibration and validation periods in ungauged case have different meaning. The results in calibration period refer to spatial transferability of model parameters since the calibration period is similar for gauged catchments as well. However, the results in validation period are much more exacting since it denotes transferability in both spatial as well as temporal domain. We implemented validation period for regionalization because it evaluates both spatial and temporal transferability.

## Results

### Model performance

**Fig. 4:**
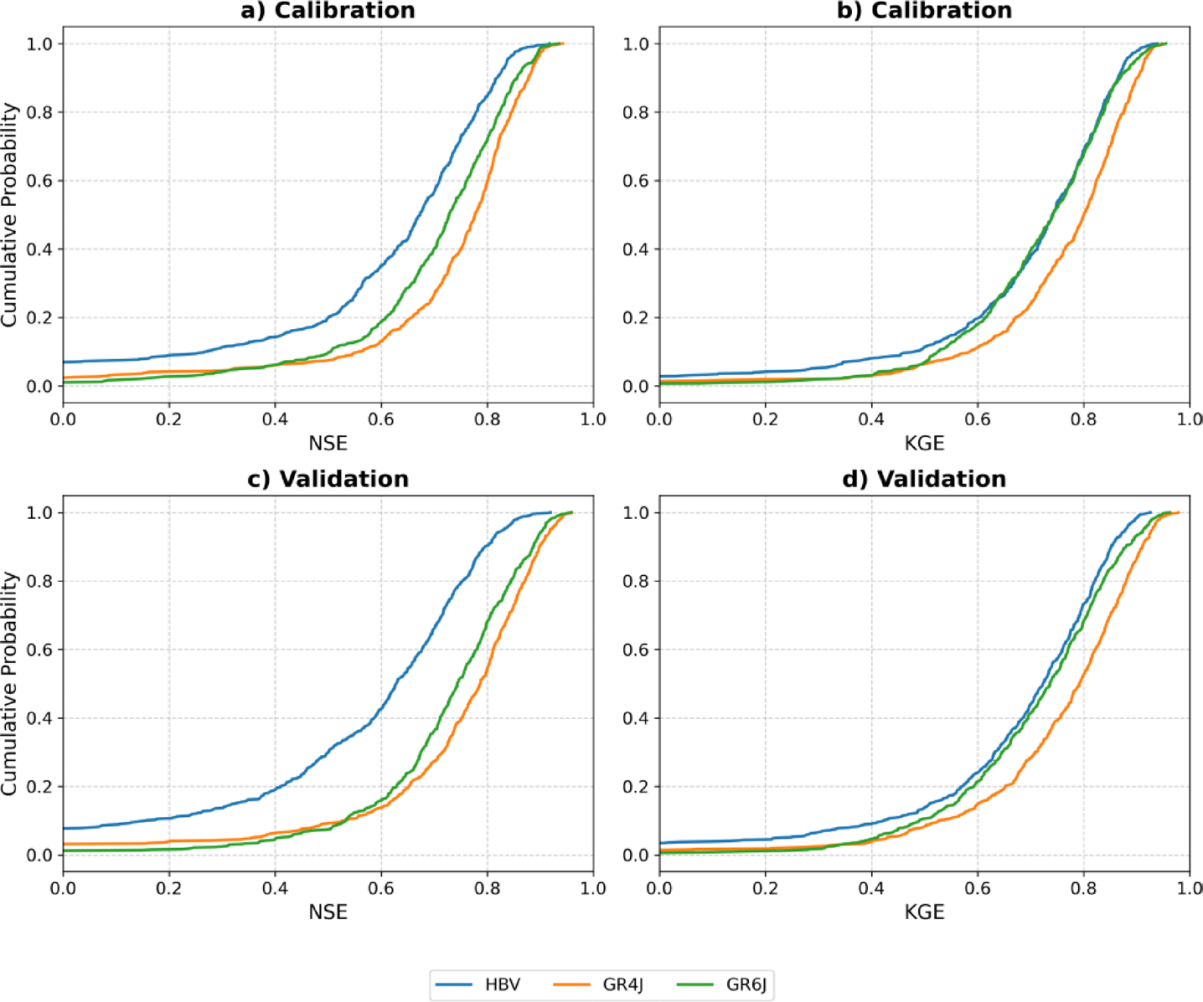
The performance of hydrological models during calibration (**a**, **b**) and validation (**c**&**d**) periods evaluated by NSE (**a**,**c**) and KGE (**b**,**d**) metrics.

Figure 4 presents the performance of models as shown by cumulative density function (CDF) curves for all hydrological models over 664 catchments measured by NSE and KGE during calibration (2000–2008) and validation (2009–2014) periods. For the calibration period, the CDF curve of all hydrological models stay close but GR4J model shifted decisively to the right of the other models across the entire distribution. This indicates that for any given probability level, GR4J achieves a higher NSE value than GR6J and HBV. Specifically, the median NSE (the value at a cumulative probability of 0.5) is the highest for GR4J. The GR6J model performs marginally worse, while the HBV model shows the lowest median performance. The range of NSE values is narrowest for the GR4J model, suggesting more consistent and reliable calibration results across the diverse set of catchments.

A similar, though slightly less pronounced, hierarchy is observed for the KGE metric. The GR4J model again exhibits the best overall performance, with its CDF curve positioned to the right. The GR6J model performs very closely to GR4J, particularly in the higher percentiles. The CDF curve of HBV model shows lower performance during calibration, which is confirmed by curve furthest to the left.

The transferability of calibrated models to an independent time period exhibits an expected decrease in performance from calibration to validation. This is a typical outcome resulting from the loss of optimality when model parameters are applied to new data. The difference in the magnitude of this performance drop highlights the robustness of model. GR4J model retains its high performance as the shift in CDF curve is the smallest between calibration and validation. While GR6J also shows reasonable robustness, its performance decline is slightly more pronounced than that of GR4J. The HBV model shows the largest decline in performance during validation as shown by CDF curve shifts left ward most significantly. The tail of low-performing catchments (low NSE and KGE values) becomes more pronounced in HBV model. This indicates that the HBV model is more prone to over-fitting during calibration and is less reliable for predictive applications in independent periods compared to the GR model.

**Fig. 5:**
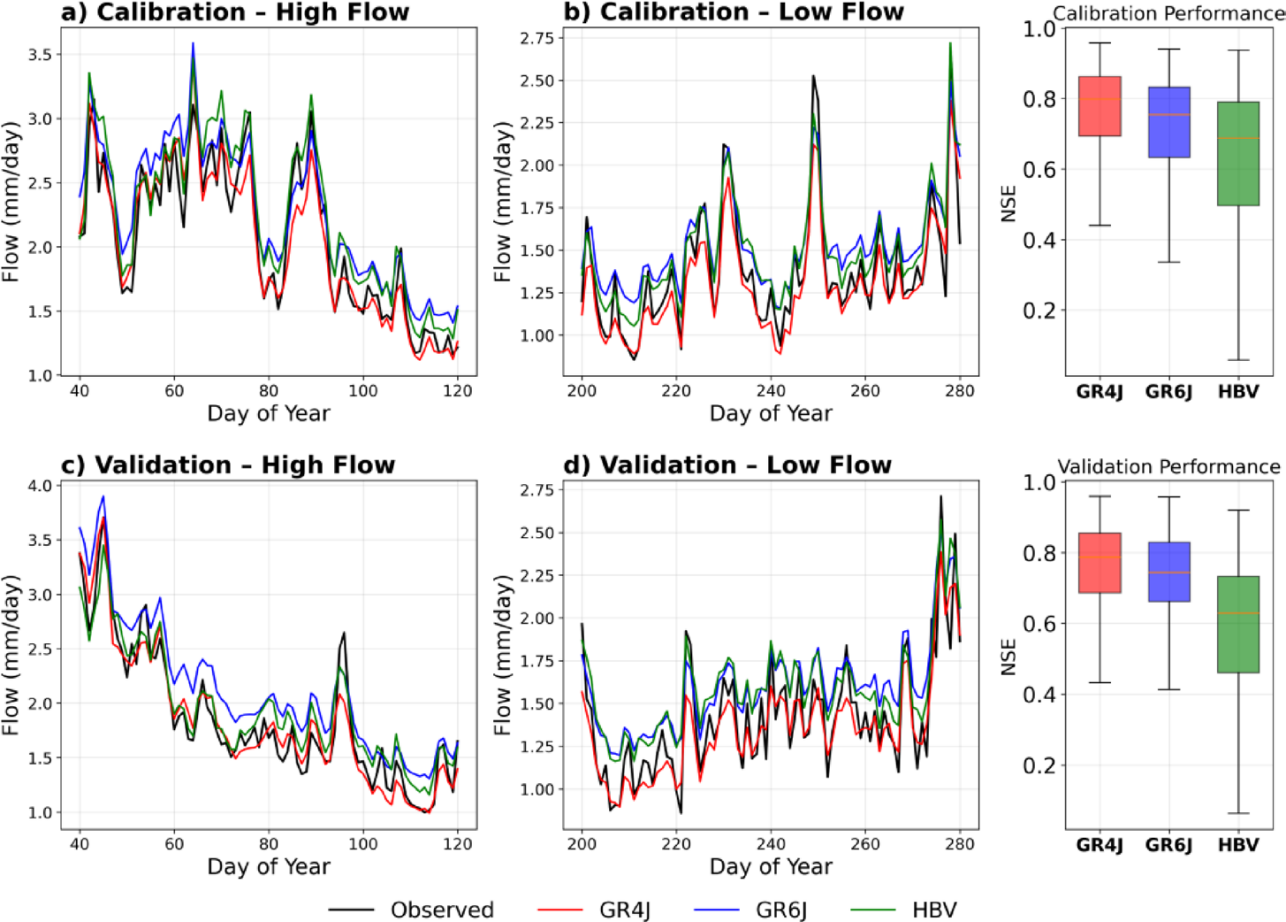
Observed and simulated daily discharge for the GR4J, GR6J, and HBV models during representative hydrological conditions. Panels (**a**–**d**) show hydrographs for calibration high-flow, calibration low-flow, validation high-flow, and validation low-flow periods, respectively. Black lines represent observed discharge, while colored lines represent simulated discharge from each model. Boxplots on the right summarize model performance across catchments using the Nash–Sutcliffe Efficiency (NSE) metric for the calibration and validation periods.

Figure 5 compares observed and simulated flows for each hydrological model averaged over the entire calibration and validation period. The GR4J model demonstrates the best overall performance for simulating mean daily flows. GR4J simulation follows the observed hydrograph with remarkable fidelity particularly during dry periods (e.g.,100 to 250 days) as well as the timing of the major flood peaks (e.g., both at the start and end of years). The simulated flow closely matches the observed flow for most of the year except slightly underestimation of the peaks. GR6J consistently overestimates observed flow particularly during the dry period (e.g., from days 100 to 250) as does the HBV simulation. The later also mostly fails to capture the intensity and sharpness of high flow events indicating underperformance in relation to flood forecasting. Additionally, the significant lag and attenuation of peaks are major drawbacks that make HBV model unsuitable for simulating the dynamic flow response. Both HBV and GR6J exhibited good performance during high-flow conditions compared to low flows, whereas GR4J maintained a more stable performance across both wet and dry periods.

GR4J outperforms the other models in the majority of catchments (e.g., 69.1% and 50.3% respectively out of 664 catchments) with the highest NSE and KGE. GR6J performs best only in 24.7% catchments based on NSE and 22.9% catchments based on KGE. HBV shows the highest NSE in only 6.2% of total catchments but comparatively performs best in 26.8% of catchments based on KGE. This shows that HBV captures flow variability better rather than absolute magnitudes. All three hydrological models fail to simulate streamflow (NSE < 0) in certain catchments. GR4J performed poorly in 22 catchments (NSE < 0) whereas GR6J failed (NSE < 0) in 11 catchments only. However, HBV exhibits the highest number of poorly performing catchments (e.g., 61 out of 664 catchments). Based on NSE results, GR4J performed best in the majority of catchments (69.1%), followed by GR6J (24.7%), while HBV achieved the top rank in only 6.2% of total catchments.

### Model performance across clusters

**Fig. 6:**
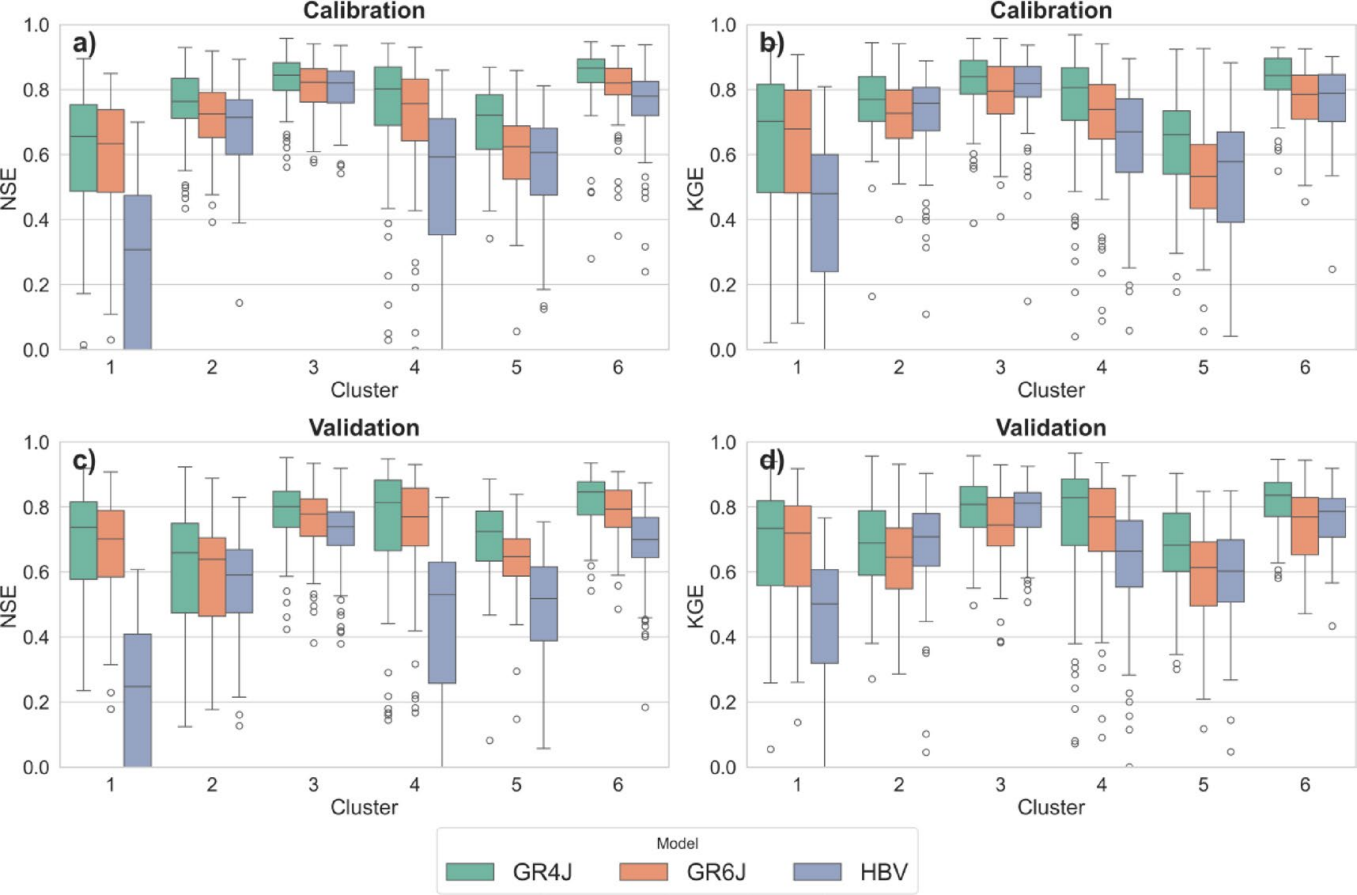
Performance of GR4J, GR6J, and HBV models across catchments within each cluster. Panels (**a**) and (**c**) show Nash–Sutcliffe Efficiency (NSE), while panels (**b**) and (**d**) show Kling–Gupta Efficiency (KGE) during calibration (**a**, **b**) and validation (**c**, **d**) periods.

Figure 6 presents the performance of each hydrological model during calibration and validation period across six clusters. GR4J and GR6J exhibited consistently higher efficiencies than HBV across most clusters in streamflow simulation. GR4J and GR6J consistently outperformed HBV across all clusters during calibration and validation periods. The higher performance of GR models (GR4J&GR6J) reflects higher streamflow simulation capability and stability in most of clusters compared to HBV. In cluster 1, GR4J and GR6J achieved good calibration (NSE ≈ 0.65–0.66; KGE ≈ 0.68–0.70) and improved validation performance (NSE ≈ 0.70–0.74). GR4J performs best in ~ 65% and ~ 54% of catchments, while GR6J performs best in only in ~ 35% and ~ 38% based on NSE and KGE, respectively. In cluster 2, all models performed well, though GR4J and GR6J slightly outperformed HBV. GR4J achieved the highest calibration efficiency (NSE ≈ 0.76, KGE = 0.77), while HBV and GR6J also showed strong but slightly reduced performance during validation. Cluster 3 showed the best overall performance, with NSE > 0.80 and KGE > 0.79 for all models during both periods. The HBV model demonstrated strong performance compared to GR4J and GR6J in Clusters 2 and 3 based on KGE values in validation period. In cluster 4, the performance of HBV declined relative to the other models (NSE ≈ 0.53; KGE ≈ 0.66 during validation). The performance of HBVwas comparable to GR6J model in Cluster 5. In cluster 6, both GR4J and GR6J achieved excellent performance (NSE > 0.79; KGE > 0.76) during calibration and validation. The modest overall degradation from calibration to validation suggests reasonable model transferability within most clusters except for Clusters 1 and 5.

In terms of model stability and variability across catchments within each cluster, GR4J and GR6J exhibit consistently strong performance in most clusters, while HBV outperforms GR models in Clusters 2 and 3 based on KGE values during validation (Fig. 6). GR4J showed stable and robust performance across clusters by maintaining similar NSE and KGE distributions between calibration and validation (e.g., particularly in Clusters 3, 4, and 6). GR6J also demonstrated relatively robust behaviour but with slightly greater variability (e.g. Clusters 2 and 5). In contrast, HBV exhibited the highest inter-cluster variability, with poor performance in Clusters 1, 4, and 5, yet notably strong and stable results in Clusters 2 and 3. These results indicate that GR4J and GR6J are generally more stable across varying hydrological conditions, while the performance of HBV is more region dependent, largely reflecting meso-climatic drivers and local hydrogeology.

**Fig. 7:**
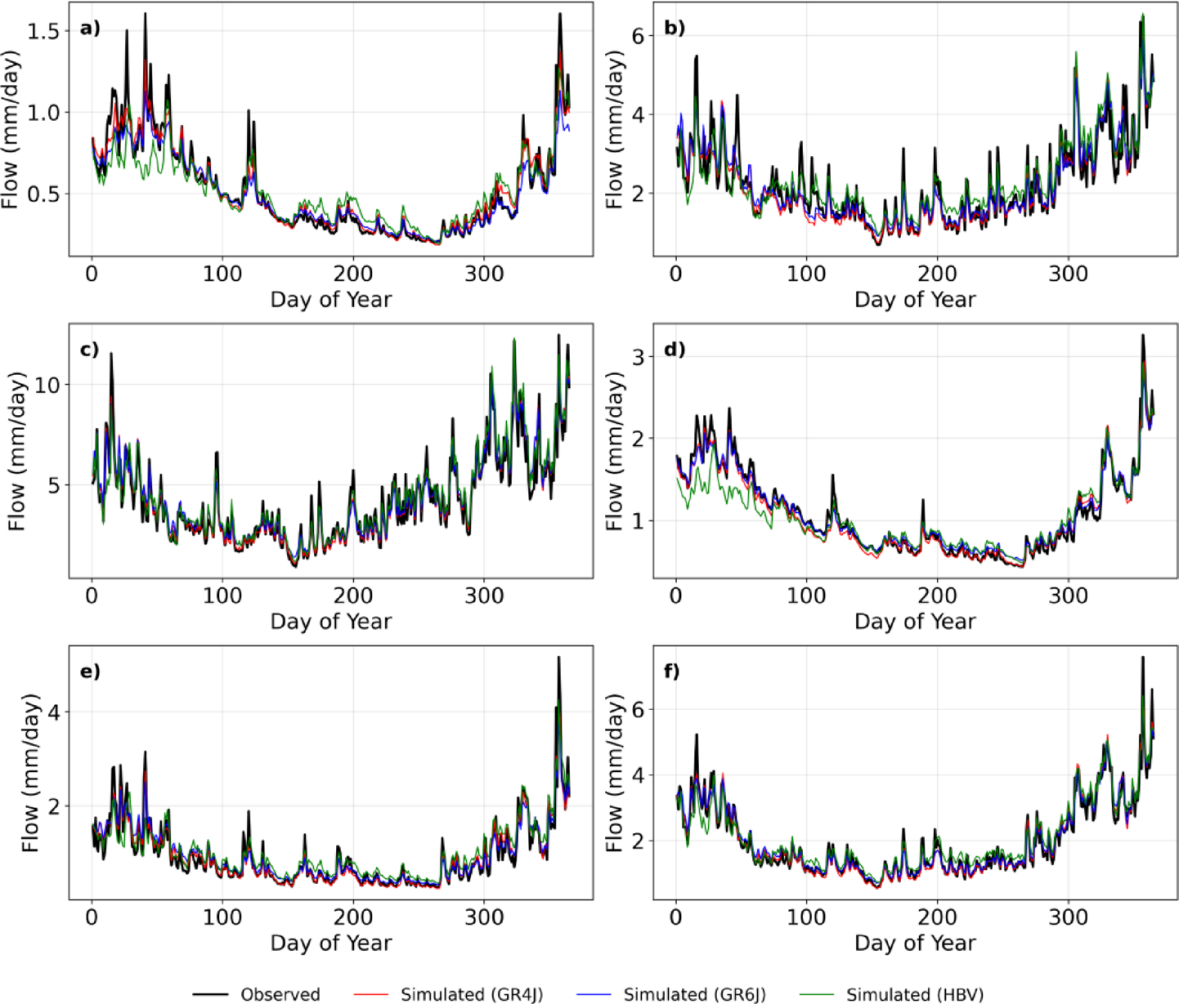
Observed and simulated daily streamflow hydrographs for catchment clusters 1–6 (**a**–**f**), showing model performance in reproducing the temporal dynamics of streamflow during the validation period.

Figure 7 presents the hydrographs for six clusters during the validation period (2009–2014) to depict the typical seasonal patterns of observed and simulated streamflow. GR4J appears to be the most visually aligned with the observed flow. It demonstrates a superior ability to capture the timing of hydrograph rises and falls as well as the magnitude of peak flows. GR6J captures the general seasonal pattern but may show systematic deviations. It struggled in accurately simulating the precise timing and sharpness of some peak flows which potentially leading to early or late peaks and an underestimation or overestimation of peak magnitudes. The HBV model exhibits a weaker representation of the flow regime and lower accuracy compared to GR models. GR models capture the observed hydrograph shape but HBV slightly overestimates low flows and underestimates winter peaks in Cluster 1 and 4.

During low flow conditions, all models show a reasonable fit but differences in the rising and recession limbs are visible. GR4J probably maintains the closest match to the observed low flows. GR6J and HBV show slight but consistent overestimation or underestimation during prolonged dry periods. Based on the visual closeness to the observed hydrograph across the six clusters, the GR4J can be tentatively ranked as the best performing model with the highest visual agreement in both timing and magnitude of flows. The GR6J and HBV models show more noticeable deviations from the observations. GR6J simulate certain flow conditions better than the HBV, but both are likely less accurate than GR4J.

### Spatial variability in hydrological model performance across Great Britain

**Fig. 8:**
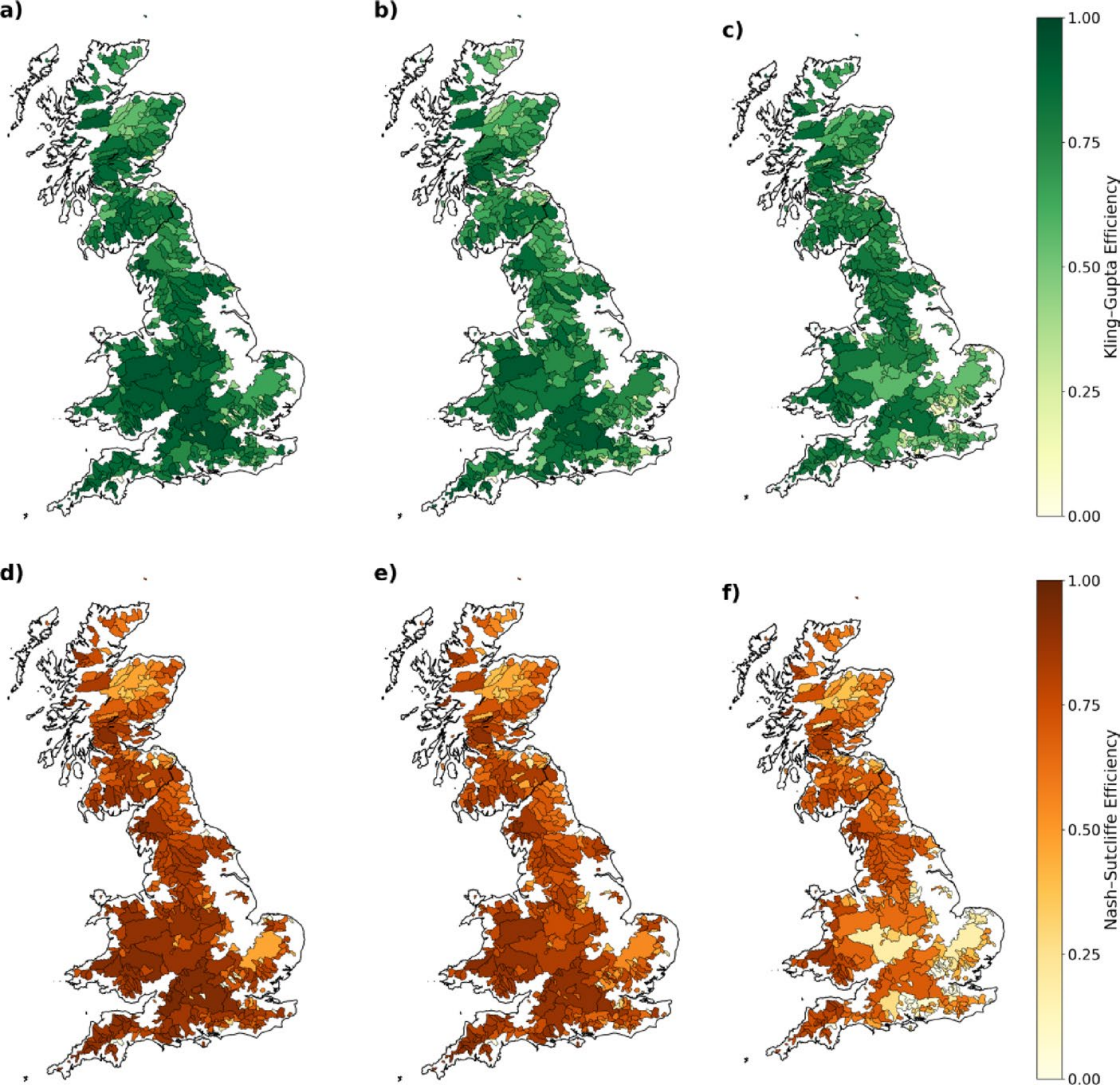
Spatial distribution of model performance metrics across the study catchments in Great Britain (GB). Upper panel (**a**, **b**, **c**) shows the Kling-Gupta Efficiency (KGE) while lower panel (**c**, **e**, **f**) shows Nash-Sutcliff Efficiency (NSE) results for GR4J (**a**, **d**), GR6J (**b**, **e**) and HBV (**e**, **f**) during validation period across UK catchments (0–1 scale; values < 0 clipped). GR4J (left), GR6J (center), HBV (right). Basins with negative values: KGE–GR4J: 11, GR6J: 5, HBV: 19; NSE – GR4J: 22, GR6J: 11, HBV: 61.

Despite its comparatively small size, GB’s diverse geography presents challenges for modelling as reflected in Fig. 8 which maps performance based on NSE and KGE statistics. In western GB (Cluster 3) all three models achieved robust simulations capturing the behaviour of this responsive group of catchments. In the eastern lowlands (Cluster 4), GR4J performed modestly but HBV was the weakest. In process terms these results suggest HBV struggles to simulate the groundwater dependent streamflow sourced from chalk and limestone aquifers.

Northern GB (Scottish Highlands; Cluster 2) has extensive upland areas higher than 1000 m ASL, which because of the northerly latitudes (57°N), translates into subarctic conditions with significant snow accumulation and melt. HBV is best suited to capturing these processes in this cluster, whereas GR4J and GR6J tend to struggle because both the latter are unable to simulate snowmelt processes, leading to poor timing. In the Central GB (Cluster 5 & 6), GR6J shows the most uniform performance while GR4J also performs well but with more scattered moderate scores. Overall, GR4J remains a robust model for responsive, rainfall-driven catchments but shows significant limitations in groundwater-dominated systems and snow-driven systems. These results demonstrates that model performance is not a single number but a geographic signature of the match between model structure and catchment processes.

### Regionalization performance and method comparison

**Fig. 9:**
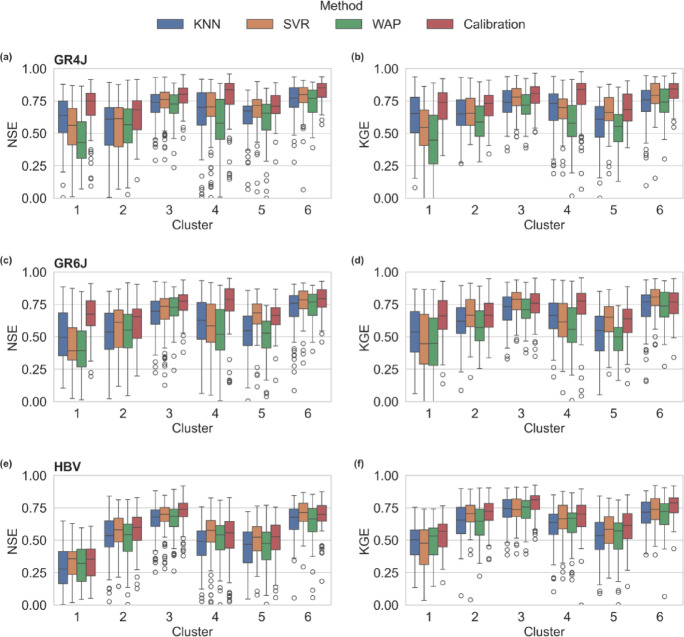
Comparison of model performance during regionalization versus site-specific calibration shown for GR4J (**a**, **b**), GR6J (**c**, **d**), and HBV (**e, f**) using NSE and KGE as performance metrics in each cluster.

Figure 9 presents a comparison of model performance during calibration and regionalization approaches using LOO-CV for GR4J, GR6J and HBV. The results show that SVR and *k*NN approaches yielded more stable results across all models and clusters compared to the WAP method. Clusters 3 and 6 showed the highest performance among all methods with minimal performance gaps between regionalization and site-specific calibration. GR4J with *k*NN or SVR achieved NSE > 0.73 in both clusters. However, Clusters 2, 4 and 5 show performance differences (NSE differences are exceeding 0.1) are dependent strongly on regionalization method and hydrological model. Among all clusters, Cluster 1 exhibited the poorest overall performance and largest method dependent variations (e.g., NSE differences between best and worst methods exceed up to 0.2). SVR approach ranked top in all clusters except Cluster 1 with all three hydrological models. *k*NN approach was consistently ranked as the second-best approach across almost all models and clusters.

In terms of hydrological model performance using the WAP approach, GR6J and HBV demonstrated better results compared to the simpler GR4J model. For example, the HBV model ranked highest (e.g., minimal performance drop between calibration and regionalization approach) in Cluster 1 and consistently ranked second in the remaining clusters (Clusters 2–5). Similarly, the GR6J model achieved the second-best performance with the WAP approach in Clusters 2, 3, and 6. In contrast, the WAP method consistently ranked third across all clusters when implemented with the GR4J model.

GR4J model proved to be the most robust model for regionalization by maintaining strong performance across multiple clusters. All models showed highest performance degradation in Cluster 1 and 4. GR4J model showed affinity with *k*NN approach in Clusters 1, 4, and 6, while responding well to SVR method in Clusters 2, 3, and 5. GR4J model shows lowest average performance drop in NSE (-0.08) using SVR regionalization compared to *k*NN (-0.15) and WAP (-0.08).

HBV exhibited higher sensitivity to regionalization approach and displayed generally lower performance metrics compared to GR models. The model responded best to SVR regionalization in most clusters (1, 2, 4, and 5). In three clusters (1, 4, 6), it even managed to slightly outperform the model with local calibration. The SVR regionalization approach effectively captures these physical relationships compared to *k*NN and WAP.

Overall, GR4J demonstrates the most robust performance across clusters for regionalization. GR6J performs similarly but exhibits slightly more variability in clusters with strong non-linear hydrological responses. HBV shows the weakest transferability, implying higher sensitivity to local calibration conditions and potentially greater parameter equifinality. Among the regionalization approaches, the SVR method emerges as the most reliable across all models.

**Fig. 10:**
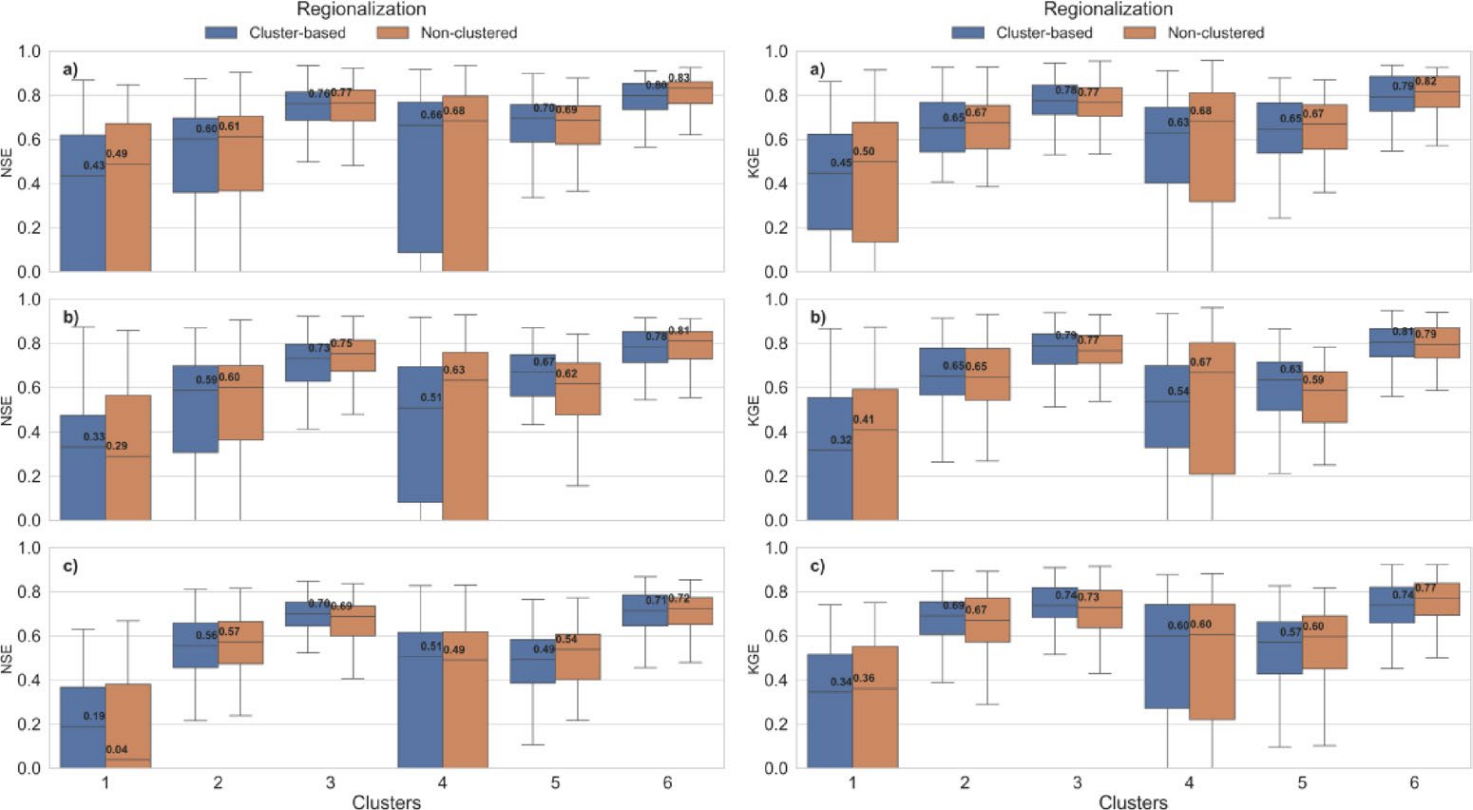
Comparison of hydrological model performance across clustered and non-clustered catchments for GR4J (**a**), GR6J (**b**), and HBV (**c**) using weighted average parameters (WAP) regionalization approach.

Figure 10 shows the overall performance of each model based on clustering and non-clustering approaches. The results shows that clustering has improved the performance of GR4J and GR6J in the WAP method, particularly in clusters with high heterogeneity, whereas HBV shows minimal differences between clustered and non-clustered catchments. This can be attributed higher structural flexibility and parameter equifinality of HBV model. SVR approach showed only marginal improvement with clustering (Fig. S1), whereas it substantially enhanced WAP regionalization by reducing heterogeneity within the donor pool. This pattern reflects the differences in regionalization methods as *k*NN operates as a local method compared to SVR and WAP. In WAP approach, the aggregation of parameters from multiple donor catchments using similarity-based weights increases robustness to donor uncertainty but also smooths inter-catchment variability. This smoothing effect proved beneficial for parsimonious GR models when combined with hydrologically informed clustering. GR models limited parameter compensation makes their performance highly sensitive to donor similarity. In contrast, the WAP approach exhibited limited sensitivity to clustering in HBV. It is most likely due to its higher parameter dimensionality and internal compensation which allowed averaged parameter sets derived from diverse donor pools to remain hydrologically functional although having potentially reduced physical interpretability.

### Performance loss from calibration to regionalization

The performance of each regionalization approach was assessed by calculating difference in NSE (ΔNSE) when moving from gauged to ungauged catchment in each cluster. Cluster 1 emerged as the most challenging region for parameter regionalization. All regionalization approaches led to substantial performance deterioration, although the *k*NN approach minimized the loss (ΔNSE = – 0.11) while lowest performance exhibited by SVR and WAP approaches (ΔNSE = – 0.32) by GR models. However, HBV model exhibited minimal performance degradation with SVR and WAP approaches. Overall, the optimal strategy remains model dependent in Cluster 1.

Clusters 2 and 3 demonstrated strong transferability of parameters using SVR approach. For the GR4J model, both the SVR and *k*NN approaches performed comparably (ΔNSE ≈ – 0.07). The GR6J model showed a clear advantage for the SVR method which exhibited only a small reduction in performance (ΔNSE = – 0.04). However, *k*NN method performed worse (ΔNSE = – 0.12) for GR6J model. SVR approach also delivered the best results using HBV model (ΔNSE = – 0.02). Given the high calibration benchmark (NSE > 0.80), all regionalization methods maintained robust predictive skill (NSE > 0.72) in these clusters.

In Cluster 4, *k*NN approach performed comparably well (ΔNSE ≈ – 0.13), while the WAP approach showed a noticeable decline in predictive skill using GR models. In contrast, the HBV model exhibited strong transferability using the SVR approach but with higher variability. Cluster 5 clearly highlights SVR approach as the most effective and reliable method. SVR approach maintained nearly identical performance to calibration (ΔNSE = + 0.01). SVR generalize better than locally calibrated ones for this cluster. Similarly, Cluster 6 shows consistently high performance across all methods. Overall, the most effective regionalization approach is SVR, although all approaches demonstrated high transferability in Clusters 5 and 6.

### Correlation between model performance and catchment attributes

The Spearman correlation analysis reveals that the relationship between catchment attributes and model performance (NSE and KGE for GR4J, GR6J, and HBV) varies substantially across the six hydrological clusters (Fig. S11). In Cluster 1, model skill is mainly associated with hydrological regime indicators, with runoff ratio, Q5, and mean discharge showing moderate positive correlations with NSE and KGE across models, while elevation and snow fraction tend to reduce performance. Land cover variables (e.g., grassland and urban) show the strongest associations, whereas hydro-climatic variables show weak controls in Cluster 2. In Cluster 3, baseflow index is the dominant factor, displaying consistently strong positive correlations with both NSE and KGE. Clusters 4 and 5 show stronger links between performance and precipitation, flow variability (Q5 and Q95), and catchment area, with negative effects from aridity and snow fraction. In Cluster 6, runoff ratio, precipitation, and Q95 exhibit the strongest positive correlations, while snow fraction, shrub cover, and aridity are negatively associated with performance. In these clusters, differences between model structures become more evident, with HBV performing relatively better in groundwater- and low-flow-dominated systems (Clusters 1, 3 and 5), while GR4J and GR6J perform better in runoff-driven regimes (Clusters 2, 4, and 6). Overall, flow regime indicators and climatic conditions emerge as the most consistent controls on model performance, with topography and land cover exerting cluster-specific influences.

## Discussion

The selection of an appropriate rainfall-runoff model to simulate streamflow remains a challenging task especially in ungauged basins^24,85^. Typically, the choice of model depends on the prior knowledge of the hydrological system, the availability of data, and experience of the analysts^18,19,86^. Earlier studies often addressed individual aspects of hydrological modelling^87,88^ while our framework integrates catchment clustering with regionalization to explicitly account for how catchment similarity, parameter transfer, and regression-based regionalization jointly influence streamflow predictions. By applying UMAP followed by k-means clustering, we achieved a more robust classification of the 664 catchments, improving upon traditional methods that relied on single-dimensional clustering or limited variables for effective regionalization^89,90^. This allows us to identify catchments behaviour in clusters and to evaluate model parameters transfer within each group. Previous studies often overlooked cluster-specific variability^91^, our results show its importance to enhance streamflow predictions. Furthermore, by systematically evaluating regionalization schemes (e.g., SVR, *k*NN and WAP), we highlighted the strength of regression-based regionalization approach in capturing watershed heterogeneity and accurately transferring model parameters to ungauged basins.

All three models demonstrate suitability for the studied catchments as indicated by satisfactory performance across distinct geographic patterns during calibration and validation (Fig. 4). Catchments in Cluster 3 and 6 exhibit similar hydrological behaviour whereas Cluster 1 demonstrates the lowest performance during calibration and validation associated with its comparatively low discharge values. Furthermore, models skill declines under regionalization in this cluster which indicate the sensitivity of model performance to parameter estimation (Fig. 8). However, the magnitude of performance degradation varies across models in each cluster due to differences in structural robustness and parameter transferability. The comparative evaluation across clusters reveals distinct differences in model robustness and transferability during calibration and validation phases. The parsimonious structure of GR4J model appears to be well-suited for capturing dominant hydrological processes while maintaining generalization (Fig. 6). GR6J remains reliable under relatively uniform hydrological conditions, particularly in Clusters 3 and 6. Similarly, Tyralis et al.^92^ reported improved performance of GR6J compared to GR4J when evaluated across a large dataset. These results emphasize that simpler, well-calibrated GR models are more robust and transferable across clusters while HBV may require additional calibration or regional parameter adjustment to maintain stability. The superior performance of simpler GR models over the relatively more complex HBV model challenges the conventional wisdom that increased complexity improves predictive capability. Similarly, earlier findings suggest that complex models are generally less stable than simpler models during the verification period^93,94^.

### Model performance and catchment characteristics

In hydrological modelling, model performance does not always show a straightforward or consistent relationship with static catchment attributes^56^. We investigated whether catchment physical attributes influence model performance, even though these characteristics were not explicitly used as inputs during model training. After generating model predictions, performance metrics such as NSE and KGE were analysed in relation to catchment attributes to assess whether certain types of catchments are associated with better or poorer model performance.

**Fig. 11:**
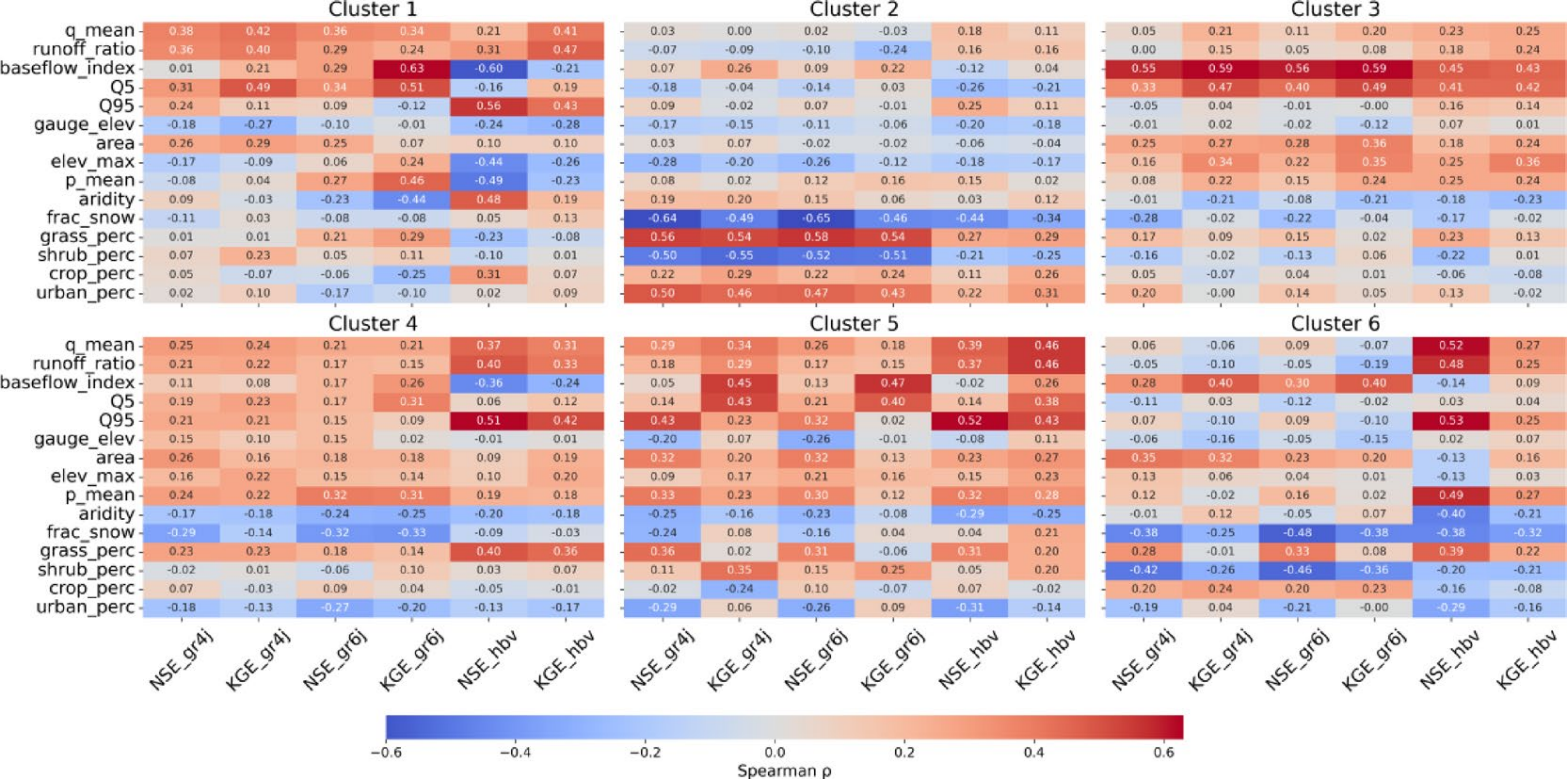
Spearman rank correlation (ρ) illustrating the relationships between catchment characteristics and hydrological model performance across six hydrological clusters. On x-axis, NSE and KGE are represented by their respective models. For example, NSE_gr4j represents NSE metrics calculated using GR4J model and so on. The abbreviations for selected attributes include mean annual precipitation (p_mean), maximum elevation (elev_max), fraction snow (frac_snow) and percent (perc) for grass_perc, shrub_perc, crop_perc and urban_perc land cover.

In Clusters 1 and 4, where cropland fraction is high (76.5% and 45.2% respectively), HBV performed poorly because it does not explicitly account for human-induced modifications such as irrigation, drainage, and soil compaction, which alter runoff generation (Fig. 11). In contrast, Clusters 2, 3, and 6 (e.g., characterized by low cropland fraction, higher mean precipitation, higher runoff ratios, and greater snow influence), enabling HBV for more accurate representation of streamflow dynamics. The negative correlation with aridity further highlights that in dry, water-limited catchments, runoff is intermittent and governed by nonlinear threshold processes, reducing parameter transferability. Compounding this, HBV parameters often exhibit strong inter-parameter correlations, leading to equifinality, where multiple parameter sets achieve similar calibration performance but diverge during validation^95^. Overall, these results suggest that parameter transferability is most reliable in catchments where physical attributes and climatic conditions are consistent with the processes represented by HBV, providing mechanistic insight into why certain clusters enable more successful regionalization. These findings align with recent work emphasizing the use of catchment attributes as proxies for dominant hydrological processes to guide model interpretability and regionalization strategies^96,97^.

In contrast, GR models showing positive relationships with discharge mean, runoff ratio, Q5/Q95, grassland fraction, and mean precipitation but the magnitude of these correlations is moderate (ρ ≈ 0.14–0.40). Their parsimonious and generalized conceptual structure does not explicitly represent many attribute-dependent processes which resulted in relatively uniform behaviour across basins^16^. According to^6^, the structure of GR4J avoids overparameterization while maintaining physical relevance, leading to more transferable parameters between calibration and validation periods. Its explicit routing components and soil moisture accounting capture catchment memory effects, supporting consistent simulation of runoff dynamics. The apparent robustness of GR models in human-modified catchments often reflects parameter compensation rather than strictly representing physical processes. Therefore, GR models perform consistently across clusters by minimizing computational errors during extreme events (Fig. 8). GR6J, with additional complexity through an extra routing store, can potentially capture a wider range of hydrological processes, but this comes at the cost of increased risk of overfitting and parameter interdependence^7^. In many catchments, this additional complexity may not be justified by the available data, resulting in reduced parameter identifiability and slightly lower validation stability compared to GR4J. Overall, these results suggest that the robust and parsimonious design of GR models favours transferability across basins and clusters, while additional complexity must be balanced against potential overfitting and data limitations. These results indicate that parameter transferability is most reliable in catchments where physical attributes and climatic conditions align with the processes represented by the model structure. This comparison highlights the trade-off between model complexity, sensitivity to catchment attributes, and transferability, providing mechanistic insight into why certain clusters enable more successful regionalization and how model design interacts with catchment characteristics.

### Evaluation of regionalization methods

There is no universal agreement on best performing regionalization approach as outcomes vary by region, model structure, and dataset size^86^. In previous studies, physical similarity^47,98^, spatial proximity^60^, and regression-based approaches^50,57^performed best based on choice of hydrological model the study area (e.g^12,36^.,, (Reichl et al. 2009)). Merz and Bloschl^48^ concluded that the spatial proximity method performed better than the regression-based approach for catchments in Austria. Similarly, Yang et al.^18,19^compared five most widely used regionalization methods using four hydrological models (GR4J, WASMOD, HBV and XAJ with 6, 8, 13 and 17 parameters). Their results show that spatial proximity and physical similarity methods performed best. In contrast, no significant differences in runoff prediction in ungauged basins was found through comparative evaluation of five rainfall-runoff models (cf^38,39,99^.). A broader synthesis by Parajka et al.^12^, based on 34 studies and 3874 catchments, that spatial proximity and physical similarity methods tend to outperform regression-based approaches. Despite the generally weaker performance of regression-based approaches reported in many earlier regionalization studies, more recent work indicates that advanced regression techniques can perform remarkably well^47,53^. These recent findings suggest that machine learning–based regression methods can rival or exceed the performance of alternative regionalization strategies in some settings. For example, regression-based regionalization approach integrated with a clustering framework outperformed both spatial proximity and physical similarity methods^57,86^. However, these studies were limited to a small set of high-quality catchments which could restrict the generalizability of their findings.

In this context, we focused on higher number of catchments and attributes to better capture spatial variability by improving clustering accuracy. By expanding the dataset, it could enhance model robustness and applicability across broader regions^60,86^. Our results by expanding the analysis to a larger spatial scale confirm that the regression-based approach remains the most effective regionalization method compared to similarity-based approaches. Our results are consistent with previous studies in UK catchments^50,100^ which show that regression-based regionalization can outperform similarity-based approaches. These results are also consistent with findings from previous studies in other regions^47,86^. Cluster 1 was the only exception, where weaker or more nonlinear relationships limited the performance of SVR approach. *k*NN remains a reliable alternative, particularly in clusters where SVR performed less well (Fig. 9). Our findings provide a direction for future research to refine regionalization strategies and enhance parameter transferability in ungauged basins.

Interestingly, our results show that the SVR regionalization approach can in some cases outperform locally calibrated models, as observed for the GR6J model in Cluster 5 (ΔNSE = + 0.02) and HBV model in Cluster 6 (ΔNSE = + 0.01). This outcome may appear counterintuitive, yet it is well supported by hydrological theory. Local calibration often captures short-term climatic anomalies or noise specific to the calibration period, leading to parameter sets that are overfitted and less transferable to independent periods. In contrast, regression-based approaches estimate parameters using information pooled from many catchments to smooth parameter variability. It can reduce the influence of equifinality to capture broader and physically meaningful relationships between catchment descriptors and model parameters. When such descriptor–parameter relationships are strong (e.g., as appears to be the case for GR6J and HBV in these clusters) the regression framework can generate parameter sets that better reflect the underlying hydrological functioning than those obtained through local calibration (Fig. 9). Consequently, regression-derived parameters may exhibit superior generalization, explaining why SVR marginally outperformed the calibrated benchmark in this part of the study. For practical water resource management in ungauged catchments, the evidence-based recommendation is to prioritize the development of regional regression relationships for the parameters of the GR4J model, as this combination provides the most reliable and transferable predictions across diverse hydrological landscapes of UK catchments.

### Cluster-dependent performance of regionalization

The cluster‐dependent regionalization results reveal a clear relationship between catchment coherence and parameter transferability, although the relationship is not strictly monotonic because different regionalization methods and model structures respond differently to catchment heterogeneity. The coherence of each cluster was evaluated by analysing the within-cluster variability of key hydrological, climatic, and physiographic attributes (Table S6). Lower variance values indicate that catchments within the cluster share more similar hydro-climatic and physiographic characteristics, while higher values reflect greater heterogeneity.

Cluster 1 emerged as the most challenging region for parameter transfer, exhibited relatively low variability in several climatic and physiographic attributes (e.g., precipitation, catchment area, and elevation). This cluster showed substantial declines in model performance during regionalization, particularly for the GR models (Fig. 9). This suggests that the similarity in broad hydro-climatic descriptors does not necessarily translate into similarity in dominant runoff generation processes. The relatively high variability observed in the baseflow index indicates differences in groundwater contributions among catchments, which may strongly influence recession behaviour and low-flow dynamics. Among the tested approaches, the kNN method minimized performance degradation, likely because it selects donor catchments based on similarity in predictor space rather than imposing a single functional relationship. In contrast, the SVR and WAP approaches showed larger performance losses for the GR models, possibly because the simpler structure of these models is less able to accommodate subtle differences in groundwater storage dynamics. The HBV model, however, exhibited minimal degradation when combined with SVR and WAP approaches, likely due to its more flexible representation of soil moisture and groundwater processes.

Clusters 2 and 3 show a stronger parameter transferability across catchments where regionalization performance improved substantially compared to Cluster 1. Although these clusters showed higher variability in some climatic and topographic attributes, the hydrological behaviour appears to be governed by more consistent runoff generation mechanisms. The SVR approach performed best across models, particularly for the GR6J model where the performance loss was minimal (ΔNSE = – 0.04). This suggests that machine learning–based parameter estimation methods can effectively capture nonlinear relationships between catchment attributes and model parameters when hydrological controls are relatively consistent across basins. The small ΔNSE values and high predictive skill (NSE > 0.72) indicate that the calibrated parameters remain representative of regional hydrological processes within these clusters. In contrast, Cluster 4 presents an intermediate case where regionalization performance varied across methods. The large variability in runoff ratio and low-flow indicators may have complicated parameter transferability due to differences in catchment storage capacity or groundwater contributions. While the kNN approach performed reasonably well, the WAP method exhibited reduced predictive skill, likely because weighted averaging may dilute parameter sets across hydrologically dissimilar catchments. The HBV model again demonstrated stronger transferability when combined with SVR, highlighting the advantage of flexible model structures in heterogeneous hydrological environments.

Clusters 5 and 6 showed highest regionalization performance due to their relatively lower variability in key hydro-climatic attributes. In Cluster 5, the SVR approach performed exceptionally well and even slightly improved performance relative to calibration (ΔNSE = + 0.01), indicating that the learned relationships between catchment characteristics and model parameters generalized effectively across basins. Cluster 5 likely contains catchments governed by similar hydrological processes and storage dynamics, allowing parameters derived from regional relationships to perform as well as locally calibrated ones. Cluster 6 also showed consistently high performance across all regionalization methods, despite some variability in catchment size. The strong predictive performance suggests that catchment scale differences did not significantly alter the dominant runoff processes represented by the models.

A key observation of this study is that success or failure of regionalization varies with hydrological characteristics of each cluster. This behaviour of cluster-dependent regionalization suggests that parameter transfer works best when the donor and target catchments share similar hydro-climatic characteristics while it performs poorly when these controls differ substantially with each cluster. Our results show that the SVR regionalization approach can in some cases outperform locally calibrated models, as observed for the GR6J model in Cluster 5 (ΔNSE = + 0.02) and HBV model in Cluster 6 (ΔNSE = + 0.01). These clusters are typically exhibited hydrologically coherent characteristics such as similarity in precipitation, seasonality, topographic and baseflow contributions.

## Conclusions

We assessed the performance of regionalization methods through three conceptual hydrological models (GR4J, GR6J, and HBV) across 664 catchments of Great Britain. By applying UMAP and KMeans-clustering approach, we identified large-scale hydrological similarities to improve model selection and regionalization for enhancement of runoff prediction in ungauged catchments. The comparative performance evaluation across six clusters highlights the joint influence of model structure, clustering and regionalization approaches on the success of regionalization. GR4J achieved the highest median NSE and KGE values in most clusters followed by GR6J. HBV model demonstrates the lowest performance during calibration and validation which highlight its limitations in regionalization. HBV performance is strongly influenced by hydrological and physiographic characteristics of catchments. The parsimonious structure of GR models provides robust and uniform predictions across clusters, whereas HBV offers advantages in specific clusters e.g. those distinguished by seasonal snowmelt.

Overall, regionalization success is strongly cluster dependent and influenced by both catchment coherence and model structure. Clusters characterized by consistent hydro-climatic controls and runoff generation mechanisms (e.g., Clusters 5 and 6) support reliable parameter transfer across catchments. In contrast, clusters with hidden process heterogeneity, particularly related to groundwater dynamics or runoff generation efficiency, can lead to larger performance losses even when basic catchment descriptors appear similar, as observed in Cluster 1.

SVR regionalization approach provided the most accurate parameter transfer and streamflow predictions for all three hydrological models (GR4J, GR6J, and HBV) across nearly all clusters. In fact, SVR regionalization performance in some clusters even outperformed local calibration (e.g., GR6J in Cluster 5) which underscores the strong potential of SVR regionalization in homogeneous or hydrologically coherent catchments. GR models showed the highest degradation in parameter transferability in Clusters 1 and 4 compared to HBV model. Although the WAP method ranked third in performance across most clusters, but its performance improved significantly when clustering was applied compared to the non-clustered approach. Our results suggest that attribute-informed parameter selection or cluster-based regionalization could improve predictive reliability in HBV model, especially in catchments with consistent, process-representative conditions. In contrast, the parsimonious structure of GR models exhibits relatively uniform behaviour across basins, yet attributes such as discharge, slope, and precipitation could help fine-tune parameters or identify basins with higher transferability. The analysis also highlights the advantage of SVR-based regionalization, which was generally the most robust method across clusters, while the performance of simpler transfer methods depended more strongly on cluster characteristics and model structure.

These findings indicate that the cluster-specific nature of parameter transferability reinforces the need for regionalization strategies that explicitly account for both hydrological model selection and the choice of regionalization method in relation to the dominant hydrological characteristics of each region.

To conclude, SVR approach offers a powerful machine learning technique for regionalization compared to conventional similarity-based approaches. The strong performance of the SVR‐based regionalization method in most of clusters demonstrates its ability to capture the nonlinear relationships between catchment attributes and hydrological responses. SVR approach can be advantageous for other regions globally where hydrological data are sparse, uncertain, or unevenly distributed. The improved performance of the WAP approach under cluster-based regionalization compared to non-clustered methods suggests that hydrologically informed clustering can enhance parameter transferability in other catchments globally. The proposed approach is transferable to data-scarce regions in facilitating parameter regionalization to enhance flood prediction and reduce potential losses from flooding.

## Supplementary Information

Below is the link to the electronic supplementary material.

Supplementary Material 1

## Data Availability

CAMELS-GB data is freely available via the UK Centre for Ecology and Hydrology Environmental Information Data Center (https://catalogue.ceh.ac.uk/documents/8344e4f3-d2ea-44f5-8afa-86d2987543a9). Hydrogr package for GR4J and GR6J hydrological models is available at (https://github.com/SimonDelmas/hydrogr). Jupyter Notebooks for model calibration and regionalization are available at https://github.com/ougahi2020/Regionalization.
