# Supplementary material for: A comparative assessment of cluster-based regionalization approaches using conceptual rainfall–runoff models
Source: Sci Rep. 2026 Apr 27;16:19982. doi: 10.1038/s41598-026-49424-z (PMC13319453; doi:10.1038/s41598-026-49424-z)
Supplement: Supplementary file 1 — Supplementary Material 1 [file 41598_2026_49424_MOESM1_ESM.docx]

A Comparative Assessment of Cluster-Based Regionalization Approaches using Conceptual Rainfall–Runoff Models

Jamal Hassan Ougahi^1,2^, John S Rowan^1^

1. *UNESCO Centre of Water Law, Policy & Science, University of Dundee, UK*
2. *Higher Education Department, Government of the Punjab, PK*

Supplementary Materials

Table S1. Calibration and validation performance of three conceptual hydrological models (GR4J, GR6J, and HBV) across six hydro-climatic clusters. Model performance was evaluated using Percent Bias (PBIAS), Kling–Gupta Efficiency (KGE), and Nash–Sutcliffe Efficiency (NSE) during both calibration and validation periods. Higher KGE and NSE values and lower absolute PBIAS indicate better model performance. The last column indicates the number of catchments in which each model achieved the best performance based on NSE and KGE.

| Clusters/ Catchments | Models | Calibration | | Validation | | Total catchments | |
| --- | --- | --- | --- | --- | --- | --- | --- |
|  |  | NSE | KGE | NSE | KGE | NSE | KGE |
| 1/94 | GR4J | 0.656 | 0.703 | 0.738 | 0.734 | 61 | 51 |
|  | GR6J | 0.634 | 0.678 | 0.702 | 0.719 | 33 | 36 |
|  | HBV | 0.303 | 0.478 | 0.247 | 0.502 | 0 | 7 |
| 2/95 | GR4J | 0.763 | 0.77 | 0.659 | 0.689 | 53 | 38 |
|  | GR6J | 0.725 | 0.727 | 0.639 | 0.645 | 24 | 13 |
|  | HBV | 0.714 | 0.758 | 0.591 | 0.708 | 18 | 44 |
| 3/140 | GR4J | 0.845 | 0.84 | 0.801 | 0.808 | 96 | 58 |
|  | GR6J | 0.822 | 0.795 | 0.778 | 0.745 | 31 | 23 |
|  | HBV | 0.82 | 0.819 | 0.739 | 0.812 | 13 | 59 |
| 4/135 | GR4J | 0.796 | 0.805 | 0.813 | 0.828 | 92 | 75 |
|  | GR6J | 0.755 | 0.736 | 0.768 | 0.768 | 34 | 39 |
|  | HBV | 0.589 | 0.669 | 0.53 | 0.664 | 1 | 13 |
| 5/95 | GR4J | 0.721 | 0.661 | 0.725 | 0.682 | 75 | 51 |
|  | GR6J | 0.624 | 0.532 | 0.648 | 0.613 | 18 | 20 |
|  | HBV | 0.604 | 0.578 | 0.518 | 0.602 | 2 | 24 |
| 6/105 | GR4J | 0.866 | 0.843 | 0.846 | 0.835 | 84 | 63 |
|  | GR6J | 0.82 | 0.785 | 0.793 | 0.768 | 19 | 18 |
|  | HBV | 0.78 | 0.789 | 0.699 | 0.786 | 2 | 24 |

Table S2 Summary table of catchment attributes in CAMELS-GB

| Attribute Name | Description | Unit |
| --- | --- | --- |
| q_mean | mean daily discharge | mm day^-1^ |
| runoff_ratio | runoff ratio, calculated as the ratio of mean daily discharge to mean daily precipitation |  |
| baseflow_index | baseflow index (ratio of mean daily baseflow to daily discharge, hydrograph separation performed using the Ladson et al., 2013 digital filter) |  |
| Q5 | 5% flow quantile (low flow) | mm day^-1^ |
| Q95 | 95% flow quantile (high flow) | mm day^-1^ |
| gauge_elev | gauge elevation |  |
| area | area |  |
| elev_max | catchment maximum elevation | m.a.s.l |
| p_mean | mean daily precipitation | mm day^-1^ |
| aridity | aridity, calculated as the ratio of mean daily potential evapotranspiration to mean daily precipitation |  |
| frac_snow | fraction of precipitation falling as snow (for days colder than 0°C) |  |
| grass_perc | percentage cover of grass and pasture | % |
| shrub_perc | percentage cover of medium scale vegetation (shrubs) | % |
| crop_perc | percentage cover of crops | % |
| urban_perc | percentage cover of suburban and urban | % |
|  |  |  |

Table S3. Summary of cluster separation metrics for different numbers of clusters (n_clusters). The table reports the mean intra-cluster distance (mean_intra), mean inter-cluster distance (mean_inter), separation ratio (mean_inter / mean_intra), and the minimum and maximum cluster sizes for each cluster configuration. Six clusters were selected as the optimal compromise between cluster distinctiveness and robust cluster sizes.

| **Clusters** | **mean_inter** | **mean_intra** | **SR** | **Min size** | **Max size** | **Interpretation** |
| --- | --- | --- | --- | --- | --- | --- |
| 2 | 12.6 | 7.9 | 0.63 | 60 | 105 | Very low separation ratio (<1): Clusters are not well-separated relative to their internal spread. Likely too few clusters; catchments are lumped together. |
| 3 | 11.7 | 8.9 | 0.76 | 36 | 67 | Slightly better, but still <1, meaning clusters are still overlapping. May capture broad differences but lacks detail. |
| 4 | 11.7 | 10.8 | 0.92 | 14 | 60 | Approaching 1. Separation improves; clusters more distinct but internal spread is still relatively high. Smallest cluster only 14 catchments → still some imbalance. |
| 5 | 10.9 | 14.5 | 1.32 | 3 | 52 | High separation ratio (>1): clusters are now well-separated. However, min_cluster_size = 3 is very small → unreliable for regionalization. |
| 6 | 11.1 | 12.1 | 1.09 | 7 | 48 | Good compromise: separation ratio >1, clusters distinct, and all clusters have ≥7 catchments → statistically more robust. Balanced max vs min size. |
| 7 | 10.4 | 13.8 | 1.32 | 3 | 45 | Highest separation ratio but smallest cluster = 3, again too few catchments → unstable for analysis. |

Table S4. Summary of cluster characteristics highlighting dominant hydrological regimes, key catchment controls, and associated implications for model performance.

| Cluster | Hydrological Regime / Profile | Key Catchment Controls | Model Performance Implications |
| --- | --- | --- | --- |
| 1 | Runoff-dominated humid systems | High runoff ratio, strong high flows (Q5), moderate precipitation, larger catchment area | GR4J and GR6J perform well due to strong runoff signals; HBV performance decreases where baseflow contributions are weak. |
| 2 | Land-cover and snow-influenced mixed systems | Strong influence of grassland, shrub, and urban cover; moderate snow fraction; weak hydro-climatic controls | All models show unstable performance; snow processes and vegetation complexity reduce model skill. |
| 3 | Groundwater-dominated / baseflow systems | High baseflow index, stable discharge regimes, moderate precipitation | All models perform well, particularly where groundwater contributions dominate flow regimes. |
| 4 | Climatically constrained mixed regimes | Moderate precipitation, influence of aridity and snow fraction, moderate catchment size | HBV performs relatively better due to groundwater representation; GR models struggle under arid or snow-affected conditions. |
| 5 | Highly variable runoff systems | Strong flow variability (high Q5 and Q95), high runoff ratio, precipitation-driven runoff | All models perform well, but performance declines with increasing aridity or urban influence. |
| 6 | Low-flow and groundwater-influenced systems | Strong low-flow signal (Q95), moderate precipitation, shrub and snow influence | HBV performs better due to low-flow and groundwater representation; GR4J and GR6J show weaker performance. |

Table S5. Summary of regionalization methods, hyperparameter settings, and training strategies used in this study. SVR hyperparameters are specified, while kNN and WAP rely on fixed design choices for reproducibility.

| Method | Key Hyperparameters / Settings | Distance / Weighting | Training Strategy | Remarks |
| --- | --- | --- | --- | --- |
| SVR | C=1.0, ε=0.1, kernel='rbf', γ='scale' | N/A | Per-parameter SVR | Target excluded in LOOCV |
| kNN | k=1 (nearest neighbor) | Euclidean (UMAP space) | Parameter transfer from nearest donor | Simple nearest donor selection |
| WAP | k=3 | Euclidean (UMAP space) | Weighted average across top-k donors | Weights = inverse distance, normalized |

Table S6. *Cluster coherence metrics based on within-cluster variance of hydrological, climatic, and topographic attributes. Key drivers of heterogeneity are highlighted for each cluster, and overall coherence (High, Moderate, Low) is provided to indicate the expected reliability of parameter regionalization within the cluster.*

| Cluster | Q mean | Runoff ratio | Baseflow index | Q5 | Q95 | P mean | aridity | Frac snow | Gauge elev | area | Elev max | Overall Coherence |
| --- | --- | --- | --- | --- | --- | --- | --- | --- | --- | --- | --- | --- |
| 1 | 0.014 | 0.134 | 1.39 | 0.14 | 0.01 | 0.017 | 0.15 | 0.069 | 0.204 | 0.03 | 0.04 | High |
| 2 | 0.18 | 0.245 | 0.427 | 0.34 | 0.24 | 0.241 | 0.17 | 2.684 | 2.236 | 0.21 | 0.72 | Low |
| 3 | 0.99 | 0.133 | 0.399 | 1.07 | 1.09 | 0.926 | 0.116 | 0.295 | 1.495 | 0.27 | 0.62 | Low |
| 4 | 0.23 | 2.001 | 0.581 | 1.75 | 0.12 | 0.055 | 0.20 | 0.138 | 0.218 | 0.41 | 0.06 | Moderate |
| 5 | 0.054 | 0.325 | 0.594 | 0.68 | 0.04 | 0.038 | 0.22 | 0.076 | 0.209 | 0.05 | 0.11 | High |
| 6 | 0.16 | 0.142 | 0.212 | 0.39 | 0.14 | 0.186 | 0.19 | 0.238 | 0.188 | 4.49 | 0.34 | Moderate |

*Figure S1. Comparison of hydrological model performance across clustered and non-clustered catchments for (a) GR4J, (b) GR6J, and (c) HBV using support vector regression (SVR) regionalization approach.*


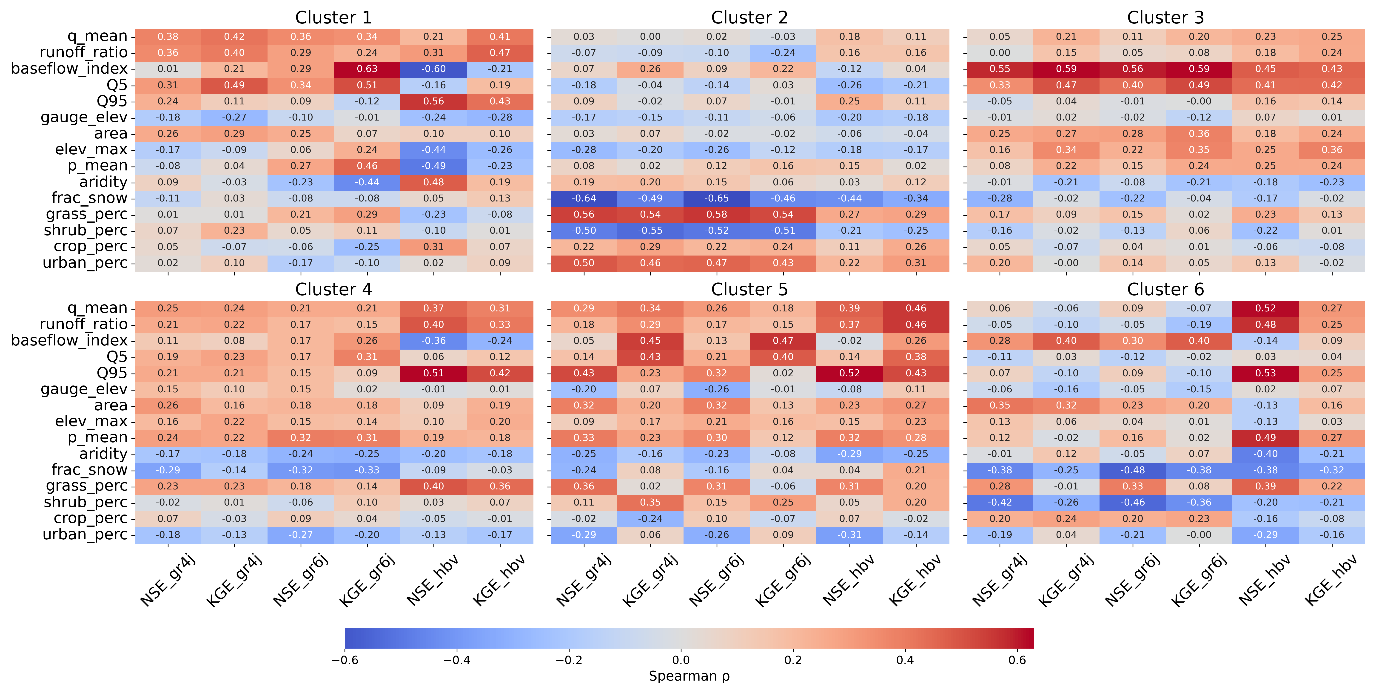

*Figure S2. Comparison of hydrological model performance across clustered and non-clustered catchments for (a) GR4J, (b) GR6J, and (c) HBV using k-nearest neighbours (kNN) regionalization approach.*
